# The Progress and Future of US Newborn Screening

**DOI:** 10.3390/ijns8030041

**Published:** 2022-07-18

**Authors:** Michael S. Watson, Michele A. Lloyd-Puryear, R. Rodney Howell

**Affiliations:** 1 Hussman Institute for Human Genomics, Miller School of Medicine, University of Miami, Miami, FL 33136, USA; rhowell@med.miami.edu

**Keywords:** newborn screening, clinical trials, genetic testing, public health, public health policy

## Abstract

Progress in newborn screening (NBS) has been driven for 60 years by developments in science and technology, growing consumer advocacy, the actions of providers involved in the care of rare disease patients, and by federal and State government funding and policies. With the current explosion of clinical trials of treatments for rare diseases, the pressure for expansion has grown, and concerns about the capacity for improvement and growth are being expressed. Genome and exome sequencing (GS/ES) have now opened more opportunities for early identification and disease prevention at all points in the lifespan. The greatest challenge facing NBS stems from the conditions most amenable to screening, and new treatment development is that we are screening for rare genetic diseases. In addition, understanding the spectrum of severity requires vast amounts of population and genomic data. We propose recommendations on improving the NBS system and addressing specific demands to grow its capacity by: better defining the criteria by which screening targets are established; financing the NBS system’s responsiveness to opportunities for expansion, including engagement and funding from stakeholders; creating a national quality assurance, data, IT, and communications infrastructure; and improving intra-governmental communications. While our recommendations may be specific to the United States, the underlying issues should be considered when working to improve NBS programs globally.

## 1. Introduction

Newborn screening (NBS) is one of the most valued public health programs in the US [1]. Through NBS, approximately 15,000 newborns are identified annually with conditions for which screening, diagnosis, and effective treatments can be used early in life to significantly impact infant morbidity and mortality. Wilson and Jungner [2] described the key features of disease screening in populations as:

*“The central idea of early disease detection and treatment is essentially simple. However, the path to its successful achievement (on the one hand; bringing to treatment those with previously undetected disease; and, on the other, avoiding harm to those persons not in need of treatment) is far from simple though sometimes it may appear deceptively easy.”*

It is over 60 years since NBS was formally initiated in the US as a State public health program to screen newborns for phenylketonuria (PKU) (see Supplemental Material and Tables S1a,b and S2–S4). Unique to the US, its State NBS programs function as 51 independent public health prevention programs. All States have specific statutes that either directly require NBS or allow for its offering to all infants born in their jurisdictions. Prominent among NBS policy and scientific accomplishments (Box 1 and Box 2) is the recognition of the development of principles upon which to base NBS actions and the need for: (1) a national scientific decision-making process; (2) the development of national and State-based quality assurance systems; (3) the development of national information systems and program standardization; (4) development of NBS information systems; and (5) development of national policy for NBS program regulation and standardization.

Box 1 **Conditions in NBS (1960–2022): Science and Technology.**

***SCIENCE* *and* *TECHNOLOGY***

- 1900, Garrod shows alkaptonuria transmits in a typical Mendelian recessive manner.
- 1900, Galactosemia, an inborn error of galactose metabolism, was first described by von Ruess
- 1934, Følling discovers phenylketonuria (PKU)
- 1949, Pauling studies molecular biology of sickle cell anemia
- 1953, Følling develops test for detecting PKU
- 1953, Bickel determines dietary treatment for PKU
- 1953, Watson and Crick elucidate structure of DNA molecule
- 1954, Maple syrup urine disease (MSUD) was first described in 1954 by Menkes et al. as a progressive neurologic degenerative disorder.
- 1960, Dancis et al. established that the metabolic block in MSUD is at the decarboxylation of branched-chain alpha-ketoacids derived from leucine, isoleucine, and valine.
- 1961, Guthrie creates first NBS test for PKU
- 1963, Galactosemia (GAL) was the second disorder found to be detectable by NBS with methods developed by Robert Guthrie and Ken Paigen.
- 1965, Thirty-two American states had enacted screening laws, all but 5 making the PKU NBS compulsory
- 1968, New York starts pilot testing newborn screening for GAL and MSUD
- 1968, Wilson and Jungner principals published.
- 1970, Forty-five states had enacted NBS laws
- 1973, Screening methods for CH and SCD developed
- 1990, MS/MS applied to NBS
- 2010, All states are screening for more than 30 conditions (many by MS/MS) in NBS
- 2012, CRISPR/Cas 9 gene editing systems discovered
- 2017, NSIGHT program demonstrates roles for genome sequencing in NBS
- 2018, First gene therapy for an NBS condition cleared by FDA: Zolgensma^®^ for SMA
- 2019, New York ScreenPlus pilot study program funded by NICHD
- 2021, Over one-hundred gene targeted therapies reported by FDA to be in late-stage clinical trials.

Box 2 **Conditions in NBS (1960–2022): Legislation, Regulation, and Policy.**

***LEGISLATION,* *REGULATION,* *AND* *POLICY***

- 1961, NICHD created.
- The Children’s Bureau of the Department of Health, Education, and Welfare and state departments of public health promoted mandatory NBS. Funded pilots/research for PKU screening.
- 1972, Sickle Cell Disease Control Act establishes SCD research centers and clinics.
- 1975, review of genetic screening and NBS by Natrional Resarch Council of National Academy of Science (NRC/NAS).
- 1976, Genetic Diseases Act was authorized to fund NIH and HRSA to establish national programs for basic and applied research and training and programs for testing, counseling, information, and education programs with respect to genetic diseases.
- 1976, Medical Devices Act.
- 1978, NSQAP created at CDC [recommendation from NRC/NAS report].
- 1978, Genetic Services program created at MCHB/HRSA.
- 1983, FDA Office of Orphan Products Development was created through the Orphan Drug Act of 1983 to provide incentives to those developing drugs for rare disorders.
- 1982, National Organization for Rare Diseases (NORD) established.
- 1983, Council of Regional Genetics Networks (CORN) established.
- 1987, NIH and HRSA convened a consensus development conference on *Newborn Screening for Sickle Cell Disease and Other Hemoglobinopathies.*
- 1987, International Society for Neonatal Screening established.
- 1989, National Human Genome Research Institute established to map human genome.
- 1993, NIH Task Force on Genetic Testing was formed. Its report in 1995 addressed the many intended uses of a genetic test from diagnosis and family genetics through population uses such as carrier screening and NBS.
- 1997, CLIAC addressed oversight under CLIA ’88 of the rapidly growing area of genetic testing.
- 1998, American Academy of Peditrics (AAP) NBS Task Force formed. Report published 2000.
- 2002, Children’s Health Act. Establishes The Secretary’s Advisory Committee on Heritable Disorders in Newborns and Children (ACHDNC) and the Heritable Disorders Program.
- 2002, Rare Diseases Act of 2002 established the Office of Rare Disease at NIH to recommend a research agenda and to coordinate related activities.
- 2002, American College of Medical Genetics (ACMG) NBS Expert Group established.
- 2003, NIH establishes the Rare Disease Clinical Research Centers.
- 2003, ACHDNC holds inaugural meeting.
- 2004-2005, ACHDNC reviews ACMG report and approves in 2005. The recommended conditions became the basis of the ACHDNC’s first RUSP.
- 2008, Newborn Screening Saves Lives Act (NBSSLA) was signed into law.
- 2009, NIH/NICHD Hunter Kelly NBS Research Program established at NIH by NBSSLA.
- 2015 NBSSLA reauthorized with new consent requirement for ‘research’ studies.
- 2015, NewSteps replaces NNSGRC as national data center for NBS.
- 2018, NBSTRN publishes recommendations for inclusion of ELSI in NBS.
- 2022, Reauthorization of NBSSLA, due in 2020, remains delayed.

Also unique to the USA are the relationships among federal agencies and between State NBS programs. In the early years of NBS, support for NBS research by the federal government and the development of State program standards and infrastructure merged federal and State public health agency efforts through support of demonstration projects and pilot studies to perform new tests/conditions. Table 1 (see Supplemental Material accompanying Table S5) views the roles of the US agencies involved in NBS.

## 2. Evolution of NBS Services, Screening Tools, Research Infrastructure and Treatment Development

### 2.1. Science and Technology

The scientific groundwork for NBS began (Box 1 and Box 2) with discoveries of a treatment for PKU in 1953 by Horst Bickel [3,4], followed by the critical development of a bacterial inhibition assay for the detection of PKU using blood absorbed onto special collection paper by Robert Guthrie [5,6,7]. Adaptations to the test made it amenable to high-volume and high throughput screening [8], making a public health program role in NBS feasible. The first demonstration project of NBS for phenylalanine was done in 1963. Successful implementation of PKU NBS led to additional assays for screening for other rare metabolic conditions [9,10,11,12,13,14], some less effectively treated than PKU. Despite automating parts of the NBS process, specimen preparation was laborious, and additional screening tests were not readily added to screening panels. Screening usually involved a singular screening test for a single disorder.

Early in the 1990s, improvements were made in automation [15], in part enabled by the introduction of electrospray ionization, sample-introduction techniques, method validation, and the development of automated interpretation systems. Concurrently, the number of specimens analyzed annually increased substantially. Technology moved from the one-at-a-time disease approach to detecting at-risk infants to multiplexed testing platforms such as tandem mass spectrometry (MS/MS) that allowed for biomarkers associated with many disorders to be detected in a single to a few analyses [16,17,18,19,20,21]. With the use of MS/MS, NBS programs greatly expanded panels between 1990 and 2010, with hearing loss (HL) becoming the first nursery-based physiological screen. Table 2 shows the decades in which all conditions now in NBS were added.

#### 2.1.1. Molecular Testing and Genomic Screening

Through the 1980s and 1990s, the specialty of medical genetics was emerging, and molecular biology allowed the development of DNA diagnostics [22,23]. At the same time, appreciation of sometimes wide variability in disease severity and penetrance grew. Over the past 50 years, specific pathogenic variants in these genes and associated phenotypes were identified, enabling genetic testing and screening [24]. However, as more asymptomatic infants at risk for a genetic disorder were evaluated and diagnosed, the clinical understanding of the complexity of genetic diseases grew.

By 2021, the etiologies of thousands of rare diseases have been established, enabling the development of specific diagnostics and treatments. To date, the molecular basis of 6925 diseases has been established [24]. A total of 4470 genes have disease-causing variants making them amenable to genetic testing [24]. The first databases of genome sequences in world populations (i.e., Exome Aggregation Database [ExAc] and the genome aggregation database [gnomAD]) have continued to develop, though they remain limited in their representations of rare pathogenic disease variants and diverse population data [25].

Currently, molecular tests in NBS are moving from functional testing with minimal result interpretation issues (e.g., TRECs) to germline testing to identify medically actionable targets. Molecular tests can be used in combination with biochemical tests as second-tier tests in the screening algorithm or to manage predictive values of screens, reduce the costs of follow-up (false-positive screens) to families and the health care system, and inform treatment in emergent neonatal situations.

#### 2.1.2. Research Infrastructure

In the US, NIH has funded several initiatives that have focused on aspects of NBS:

1. The National Human Genetics Research Institute (NHGRI)/NICHD-funded Newborn Sequencing in Genomic Medicine and Public Health (NSIGHT) program demonstrated the potential roles for exome sequencing (ES) or genome sequencing (GS) in NBS [26,27,28]. Among cases screening positively for an inborn error of metabolism (IEM) by traditional NBS methods across two study sites, specificity was 94%, and 86–88% of newborns were detected (clinical sensitivity) by ES/GS. [26] Much of the reduction in clinical sensitivity resulted from the proportion of cases with variants of uncertain significance (VUS) that aren’t reported when used for screening of asymptomatic people. Both study groups considered this performance and result turn-around-times to be inadequate to replace traditional NBS methods with ES/GS at this time. The potential for ES/GS to contribute to NBS for non-IEM disorders is apparent in early-onset HL [29]. The number of infants with HL not detected at birth but likely to realize a benefit from early treatment similar to that detected by NBS audiometry is nearly equal to the number of infants found by traditional HL NBS [29].
2. Simultaneous with the NSIGHT projects, NHGRI and NICHD funded The Clinical Genome Resource [30] that prioritized NBS and genomic screening genes among its gene and variant clinical curation activities to minimize uncertain findings as new screens are implemented. Genomic screening emerged through consented reporting of medically actionable secondary findings (SF) [31] that could be screened over the lifespan, including in NBS. More recently, metabolomic profiles (the complete set of small-molecule (<1.5 kDa) metabolites) have shown potential for IEM screening in NBS [32].
3. NICHD funded the Newborn Screening Translational Research Network (NBSTRN) through a contract with the American College of Medical Genetics (ACMG) to establish and operate it as a bridge between research and clinical investigation to enhance the knowledge base and clinical care, and to develop the tools to support large multi-State NBS pilot studies [33].

#### 2.1.3. Developing a Treatment Pipeline

The pipeline for new drugs and biologics is filling after developing rapidly over the past decade. The first gene therapy for an NBS condition, Zolgensma^®^, became available shortly after Spinraza^®^ was cleared by the FDA and led to the addition of SMA to NBS. In 2020, the first patient with SCD was treated using clustered regularly interspaced short palindromic repeats (CRISPR) Cas 9 gene replacement therapies. Even though these treatments are for rare disorders, concerns were also raised about treatments becoming cost-prohibitive as, for example, Zolgensma’s^®^ costs were approximately $2.2 million for a one-dose treatment.

#### 2.1.4. Pilot Studies of Candidate NBS Conditions

Pilot studies in an unbiased general population to generate the data needed for decision-making are among the last of the premarket stages of investigation of conditions considered to be candidates for addition to NBS. Population screening increases understanding of the disease and its spectrum of severity. NBSTRN became active in collaborating with State NBS programs and investigators on population-stage pilot studies of Pompe disease, mucopolysaccharidosis type II (MPS II), spinal muscular atrophy (SMA), and X-linked adrenal leukodystrophy (X-ALD), and Duchenne muscular dystrophy (DMD) [34,35,36,37,38,39,40,41]. One of the more robust NBSTRN pilot studies that evaluated screening for a condition was done by NBSTRN for severe combined immunodeficiency (SCID) disorders [39,40]. It highlighted the difficulty in obtaining sufficient statistical power with rare disease studies, even with seemingly very large multi-State populations, and the need for extended follow-up data collection, particularly when one of the multiple conditions of varying prevalence is among the possible diagnoses [39].

The NBSTRN pilots also pointed to the need for improved data collection infrastructure. NBS historically includes short-term follow-up (STFU) and long-term follow-up (LTFU). STFU includes establishing a diagnosis, or not, and the plans for or the initiation of treatment. LTFU includes the initiation of and response to treatment, connection to related services, and clinical outcome evaluations to assist in system quality improvements. The clinical outcomes are particularly important in establishing clinical validity and utility [42]. Ongoing data collection and analyses are essential in supporting the continuation of screening, diagnoses, and treatment, and for understanding whether the expected screening outcomes have been realized.

Data from STFU and LTFU of screen-positive newborns has highlighted how biased our views of genetic diseases may be when cases are ascertained through studies of those clinically affected and their families. Unbiased ascertainment typically results in a better understanding of disease incidence and identification of a broader range of disease severity, particularly at the mild end of the disease spectrum. Full population screening, as in NBS pilots, identifies later-onset forms of disease and provides a less biased estimate of disease penetrance.

Alternative approaches to clinical trials of new NBS conditions outside the public health system are now emerging. Examples include the New York Screen Plus pilot study program funded by NIH [43] and hospital-based screening, and as another example, by Parad et al. for DMD [44]. The availability of standardized criteria from ACHDNC by which conditions being considered for addition to NBS are assessed provides a useful framework on which to build [45].

### 2.2. From Developing Guidelines to Implementing Legislation

Significant to NBS, the roles of government, families, and health care professionals have been critical to developing NBS programs and their support, especially regarding policy development (Box 1 and Box 2). Because NBS was increasingly focused on genetic diseases, the first recommendations from a nongovernmental organization (NGO) were on genetic testing, including aspects of NBS that brought a specific focus to NBS and rare diseases. The NRC report [12] was prophetic in making the following statement: “As new screening tests are devised, they should be carefully reviewed. If the experimental rate of discovery of new genetic characteristics means an accelerating rate of appearance of new screening tests, now is the time to develop the medical and social apparatus to accommodate what may otherwise turn out to be unmanageable growth”. Consensus conferences concluded that universal screening for SCD should be provided [46,47]. Other recommendations for NBS came from the American Academy of Pediatrics (AAP) in 1999 [46], the Institute of Medicine (IOM) with Assessing Genetic Risks in 1998 [48], and ACMG) in 2005 [49]. The various commissioned groups’ recommendations have formed the basis of legislative action implemented over time.

Now there are federal agencies in existence, responsive and responsible for carrying out the programs and supporting research on various aspects of genetics and NBS, including implementing a federal law that protects consumers from discrimination by their employers and the insurance industry based on genetic information (Table 1). The AAP recommendations became the basis of the Heritable Disease Program enacted under the Child Health Act, 2000. This Program authorized the formation of the Advisory Committee on Heritable Disorders and Newborns and Children (ACHDNC), and the Program was further refined under the Newborn Screening Saves Lives Act, 2008. Among its first acts was endorsing the uniform panel proposed by ACMG [49] designated as the Recommended Uniform Screening Panel (RUSP). NBSSLA provided an administrative structure (the Interagency Coordinating Committee [ICC]) to organize federal agencies around NBS and genetics, but there was no clear administrative structure intersecting federal and State public health programs, specifically NBS and genetics. A government-wide assessment by the Government Accountability Office (GAO) also pointed to the lack of federal and State coordination [50].

Government policies also affect the ability of families to access treatments. Analysis of birth and death data over the period of the institution of Medicaid and the timing of NBS mandates show that NBS is associated with improvements in infant mortality in States with Medicaid [51]. And in contrast, NBS mandates were not linked to significant declines in infant mortality in States without Medicaid, indicating the importance of access to health care and treatments.

## 3. Newborn Screening in 2022

Currently, State NBS programs are experiencing pressure to consider: (1) RUSP expansion, (2) the potential benefits and harms of screening for new conditions, and (3) how to integrate genomic screening into the population at any age, including in newborns. Within this context, and along with the current status of public health and health care infrastructure, several challenges can be identified.

### 3.1. Lack of Infrastructure to Acquire Data for Studying Rare Diseases

#### 3.1.1. Information Technology Needs

Disease rarity remains a central obstacle to acquiring statistically robust data to support nominations for addition to the RUSP and confounds the decision of when a pilot study has satisfied the analytical and clinical validity questions. While knowledge about those clinically affected by these rare genetic diseases is improving within clinical practice, understanding the full range of disease severity and penetrance remains limited due to a lack of general population data. The lack of general population data distorts the birth prevalence data and the clinical history of these conditions. NBS, through population-based screening, allows the accumulation of information that can clarify aspects of genetic diseases that complicate their transition from diagnostic uses to uses in screening in asymptomatic individuals (in either NBS or another clinical setting).

Data accumulation is further hampered by the lack of IT. As evidenced by the inability to share the percent immunized or not immunized, or who require hospitalization for COVID-19 or share their clinical history, similar constraints to data sharing remain for NBS in the USA. IT communication tools were introduced in the 1980s when the 1st fax machines and microcomputers became available, leading to improved communications and result delivery between the NBS program and the health care delivery system. However, there has been little movement in developing robust health IT systems beyond recognizing need [52,53]. More than one-third of local US health departments reported their inability to access an electronic surveillance system with data from local emergency departments, which could facilitate the early identification of screen-positive infants identified through NBS [52,53,54,55,56]. In addition, no national standards exist for performance characteristics (e.g., false-positive rates, predictive values of screening tests) of either pilot studies of candidate conditions for NBS or for currently mandated conditions.

#### 3.1.2. Research Funding

In the USA, multiple approaches have been taken to fund rare disease research, clinical investigation, and practice [26,27,28,30,33,57]. However, these approaches rarely have included the breadth of participation needed to address the disease performance goals of NBS and rarely recognize the need for collaboration with State NBS programs. Current funding programs include those listed in Table 3.

### 3.2. Standards for Transition to Inclusion of Molecular Technology

Evolving molecular technologies such as long-read sequencing are filling in the gaps in the human genome sequence that account for approximately 15% of the genome in the 2004 ‘more complete’ genome sequence [58]. More than 100 genes and millions more variants were found. As of 2021, there are 73 genes on the ACMG’s secondary findings (SF v3.1 list of medically actionable genomic targets, most related to cancer and cardiovascular disease predispositions, but the list includes NBS genes for which actionable juvenile or late-onset adult forms are well described [59,60].

Molecular testing platforms capable of detecting multiple analytes, such as gene/variant panels and next-generation sequencing (NGS), are now commonly used in the 2nd tier of NBS testing algorithms and diagnostic follow-up. Sequencing a newborn’s genome could provide more health information than the current panel of tests. It could potentially be used to guide an individual’s lifetime of medical care, providing early information on both treatable childhood diseases and conditions that occur in adulthood.

However, genomic screening is early in its development, with many of the same rare disease problems as seen in NBS. In addition, it is being used over the lifespan of an individual. There has been limited data from cases ascertained unbiasedly in the general population and a vast array of medically actionable conditions for which risk assessment is possible after the screening test’s performance characteristics have been established. High positive predictive values (PPVs) will be needed to minimize the high costs of laboratory and clinical follow-up. At the other end of the spectrum are private variants so rare as to preclude comparison with other cases but for which a therapy tailored to the variant is becoming a reality. If we are to take advantage of the opportunities developing for the identification and treatment of medically actionable rare and ultrarare genetic diseases, for which genetic variants may be the only biomarkers available, early genome sequencing will be needed.

As data accrues on the clinical significance of rare variants that lack specificity on their associated disease severity and age of onset, some variants may be shown to predispose to a late-onset or attenuated form of a disease that may not have been the goal of NBS unless treatable early in life. Experience has shown that certain patients with late-onset Pompe disease develop symptoms requiring treatment in childhood. To maintain the NBS focus on identifying infants with conditions treatable early in life, either improved functional assays that clarify the pathogenic nature of a variant or large bodies of data that inform this question should allow for the adaptation of the screening algorithms. Nevertheless, the key measure of a condition’s appropriateness for NBS is the outcome of the subset of screen-positive cases treated during clinical trials leading to improved outcomes.

As genome screening evolves, some genes/variants/conditions will likely meet the requirements for NBS. Private laboratories can participate in NBS though under contractual agreements with NBS programs which bring them under the public health programs’ rules and exemptions under privacy laws. There are currently four private entities (Genome England, SEMA4, Perkin Elmer, and Fulgent) with NBS gene panel tests on the market but outside of NBS programs. However, there is substantial variability in their genome targets, suggesting that industry standards are needed [61]. Genomics England is now planning to take advantage of the UK’s national health data to engage a 200,000 infant pilot study of the use of GS in NBS [62].

### 3.3. Delays in Therapeutic Development

The often-long lag times between gene discovery and treatments are yielding to the growing development of drugs for rare genetic diseases. In addition, many new treatments in development are specific to a patient’s molecular status, which is usually an even rarer event than the disease itself. Some treatments are being assessed in clinical trials for conditions in which an entire gene or a more common gene variant can be targeted, while others are being made available for ultrarare disease situations under compassionate care constraints. The rapidly growing list of new drugs has significant implications for NBS since effective treatment is often the main impediment to adding a condition. Orphan Drug Designations at FDA increased by 41% from 2019–2021 to 753. Rare Pediatric Disease designations increased 330% from 2019–2020 to 284. FDA has received more than 900 investigational new drug (IND) applications for gene therapy clinical studies; FDA expects 200 INDs per year beginning in 2021 though safety profiles and durability of response remain an obstacle [63]. As of February 2021, there are 109 late-stage gene-targeted therapies. By 2025, FDA expects to be approving 10–20 cell and gene therapies per year [63]. New treatment modalities are of several types, including molecularly targeted drugs that allow in vivo gene editing [e.g., CRISPR, antisense oligonucleotides (ASOs), messenger ribonucleic acid (mRNA), small interfering RNA (siRNA), endless RNA (eRNA)] and in vitro gene replacement therapies [e.g., adeno-associated virus (AAV)]. However, the manufacturing of many of these treatments is highly complex, straining manufacturing and production capacity, as evidenced by recent COVID-19 vaccine manufacturing [64].

Due to the rapid development of new therapeutics after a decade of slow expansion of NBS, the candidate conditions list for NBS has grown. The NBSTRN identified 15 candidates for NBS pilot studies through an expert opinion survey of medical geneticists and metabolic disease physicians [65]. More candidates for NBS are entering the research and development pathways tied to new treatments in clinical trials. The NBSTRN is currently collaborating on pilot studies of Duchenne, Krabbe disease, GAMT deficiency, and MPS II, although standards by which success of a pilot study is measured remain ill-defined.

The growing number of candidate conditions for NBS is not aligned with the public funding available for either pilot studies or ACHDNC reviews of nominated conditions. Recognizing the need for pilot studies, lay advocacy groups, private organizations partnered with States, and pharmaceutical companies have partnered to establish NBS pilots. Initially supported by Biogen for an SMA pilot, the NBS clinical trial and NBS pilot infrastructure in New York was followed in 2018 by multiple pharmaceutical companies supporting a pilot study of DMD in NY [66].

### 3.4. Delays in State Implementation of RUSP

Implementation of RUSP additions remains long and uneven. Reports at the September 2019 ACHDNC meeting [67] showed that it can take 3–10 years to implement a condition in all States after HHS has recommended addition to the RUSP, often after a long period of pilot testing to develop the needed clinical validity data. SCID and CCHD were recommended in 2010 and took 10 and 9 years, respectively, to be implemented in all States/territories; Pompe, MPS I, and X-ALD were recommended to RUSP in 2015–16 but remain to be implemented in 55% of State NBS programs.

## 4. Preparing for the Future: Challenges and Solutions

We focus on the emerging aspects of NBS likely to contribute to the solutions to growing program needs. Some aspects are US-specific, while others also will be faced in other countries. These include increasing capabilities and capacity in information technology (IT), informatics/bioinformatics, and communication; strengthening and partnering with disease support groups; strengthening the research infrastructure; and educating and training the broad workforce engaged with NBS. All are central to how the NBS community and system acquire the necessary skills, capacity, and infrastructure to digest and assimilate the wave of screening technologies and treatments in development. While our recommendations are often specific to the United States, the underlying issues should be considered when working to improve NBS programs globally.

The COVID-19 pandemic has revealed a public health workforce in crisis. Nationally, all local health departments employ an estimated 153,000 workers, down from more than 184,000 before the recession of 2008. Few public health departments have staff such as community health workers, epidemiologists, statisticians, or public information professionals in specialized roles critical to delivering essential public health services. These gaps are even more acute in rural areas, where many health departments struggle to maintain the provision of safety-net health care services [55,56].

Although IT and data capacity are key elements of public health capacity, much of State and local public health work has changed little between the 1975 NAS report [12], the ACMG report in 2006 [49], and the recent report by Bailey et al. [68]. Our healthcare system still lacks access to an electronic surveillance system with data from local emergency departments that could facilitate early identification of screen-positive infants identified through NBS, and few local health departments have interoperable systems. Complicating this picture further is a lack of systematic collection of data in critical areas, such as data on race and ethnicity, needed to track disparities and assess equity [69,70].

Finally, there is a dearth of financial resources. Although there is no systematic accounting for all relevant spending, it is clear that public health in the United States has been chronically underfunded. In addition to gaps in support for specific federal health efforts such as pandemic preparedness now exacerbated by COVID-19, State government funding for public health has stagnated, with no growth occurring between 2008 and 2018 [71,72]. The recommendations below address several areas needing improvement to enable the effective expansion of NBS systems. The rapid pace of evolution of science and technology is faced by many high-income countries while we discuss policy and legislation in the context of the US.

Recommendations: Five Major Areas for Improvement

- Criteria for newborn and child screening
- Financing of the NBS system
- National quality assurance infrastructure
- Data and IT communications Infrastructure.
- Intra-government communications.

### 4.1. Criteria for NBS Newborn and Child Screening

#### 4.1.1. Which Conditions to Include in NBS?

It is difficult to compare individuals with rare genetic diseases because they differ in incidence, severity, age of onset, penetrance, and response to and risks of treatments. The variable spectrum of disease expression has left unclear endpoints that define when enough is known about screening for a candidate condition to formally evaluate its performance through national bodies such as the US ACHDNC for possible inclusion in the screening panel. Further, the nature of the benefits that justify NBS for a condition and to whom they should accrue are being challenged because genetic disease inheritance implicates not just an infant but its nuclear and extended family. Genetic screening might be appropriate, even when a direct medical treatment is not available, if there is a benefit to the baby through management and support to the family, to inform subsequent reproductive decisions, and to provide society with knowledge about the condition. Initially, ACHDNC captured benefits for the family within its nomination and review criteria, but since 2012, benefit to the family has been removed from consideration.

We, therefore, recommend a re-evaluation of the criteria for inclusion of conditions in newborns as follows:

- Redefine the primary targets of screening to be the forms of a disease that are treated during the pilot study and, thereby, inform the initial clinical validity determination of including a particular condition in the RUSP.
- Reevaluate the benefits that justify NBS and consider other potential benefits, including whether detection of genetic carrier status is a reasonable goal of NBS.

#### 4.1.2. Newborns and Children: When to Screen?

NBS is unique as one of the very few times in life when there is access to the entire population. Other opportune times for childhood screening include the age at which an infant receives most of their immunizations, either as infants or prior to entering school or as adolescents.

Conditions requiring very fast result turn-around-times and treatment initiation for the health of the infant raise the question of whether case-finding and intervention should be through public health programs or included in a growing number of neonatal or pediatric health care services offered as standard of care for infants (e.g., hyperbilirubinemia leading to kernicterus), children, or adolescents.

For disorders that may not require treatment until well beyond the adolescent period making the availability of electronic health records (EHRs) would document a patient’s need that could be met in the future by public health and/or private health care providers. Additionally, as knowledge of the molecular etiologies of the disease improves, the role of genome sequencing in screening over the lifespan, including in NBS, will grow. Other disorders may not require treatment until well beyond the adolescent period making the availability of EHRs critical to documenting a future patient need that could be met by public health and/or private health care providers.

Pediatric care providers are usually required to report immunization completions to State registries which allows linking the registries to screening databases. Incorporating physiologic screening methods or point of care screening tools used in such a clinical setting could also be considered. However, there may be reduced universal coverage, and not all infants may be detected.

Screening targets could include those not as robust in the first 24–48 h of life, such as for HL [29]. For example, rescreening for HL could double the number of cases identified early enough to benefit from HL interventions [29]. Some conditions currently recommended for NBS may have prenatal onset, as with in utero neuronal loss in SMA, suggesting that prenatal screening with cell-free DNA (cfDNA) would have greater clinical benefit if proven prenatal treatments exist.

Ultimately, the life stage at which screening may be appropriate will depend on the consensus of when treatments should start to maximize the benefit to those affected.

We, therefore, recommend that decision-making bodies such as ACHDNC in the US should:

- Work with pediatric health care providers to coordinate an approach to screening over the life course, identifying points of intersection between various child health screening programs.
- Work with medical and public health organizations, specialty societies, families, and EHR registries to develop IT tools that public health and health care providers can use to address interoperability to track and that families can use as a “health profile” passport.
- Integrate investigative efforts to implement genome screening over the lifespan as NBS evolves.
- Consider whether any candidate conditions for genomic screening meet the criteria established for NBS.

#### 4.1.3. Flexibility in the Nomination and Review Processes

Unique circumstances may suggest alternatives to one-at-a-time nominations of conditions to the RUSP. The initial US RUSP recommendation was based on the ACMG’s expert review of a large number of genetic conditions considered to be candidates for NBS. It excluded infectious diseases, such as CMV, for which NBS remains variable among the States. The expanding number of clinically significant globin alleles points to the need to move beyond conditions defined by an S allele. A prime target of unique pilots could be the subset of conditions not on the RUSP but for which analytical data on specific biomarkers are available from routine screening for other RUSP conditions.

- ACHDNC should consider commissioning large reviews of groups of conditions, including infectious diseases and hemoglobinopathies.
- Funding bodies (e.g., the NICHD in the US) should develop funding for clinical (LTFU) data collection when analytical screening data is already available from prior screens.

### 4.2. Financing Newborn Screening Systems

Pilot studies of the rare and variable conditions being considered for addition to NBS face significant challenges as effective treatments for more conditions become available. As those conditions become candidates for NBS, the costs of keeping NBS panels current will increase. Financing needs to consider: (1) the infrastructure needed to ensure that pilot studies include large and geographically diverse populations is lacking, (2) the standards by which analytical and clinical performance of newborn screens are judged and overseen are limited, and (3) funding available for the high costs of pilot studies and central review is limited. Thus far, a disconnect remains between the State’s investment in NBS and the return on investment that accrues to both the US and State’s public financing programs, the private health care system, families, and the industries that meet the needs of NBS programs and patients. Furthermore, financing has not kept pace with NBS expansion.

#### 4.2.1. Costs of Screening

When developing financial plans for NBS programs, the cumulative cost of screening processes, education, and diagnostic follow-up must be considered. The two predominant methods by which State NBS programs are funded are general revenue from the State that includes federal support and fees for service. In the short term, funding and collection of service fees are available but vary by State, and not all States have NBS fees [50,73,74,75]. And the fee doesn’t necessarily include all costs, especially considering the growing need for research and development. Public-private partnership funding also is increasingly being used to fund NBS equipment and pilot studies. Determining the cost of screening is a multi-dimensional decision process. It typically includes an assessment of the offset by longer-term cost savings through reduction of acute care costs to families and society, and the increased productivity of those identified and treated.

- Continuous review and updating of NBS financing programs are needed to ensure that the entire system keeps up with advances in NBS with a focus on programs that equalize access to services and treatments.
- A sustainable and manageable funding system, such as direct billing to hospitals as exists in some States, should be considered as a national plan.
- ACHDNC, in its capacity as advisory to the Secretary of HHS, should provide expert advice regarding how best to distribute the costs and benefits of NBS to avoid unfunded mandates.
- Federal Medicaid reimbursements to States should require a standardization of how funds for NBS are utilized in all States (currently, each State can decide independently whether and how much funding can be used and in what manner).

Drug approval for treating rare diseases is the most rapidly expanding area of drug development. Because of the small numbers of patients who suffer from each disease, the FDA often allows nontraditional approaches for establishing safety and effectiveness, for example, the use of smaller, nonrandomized, unblended trials and use of surrogate clinical endpoints. The benefits from the explosion of new therapeutics won’t maximally benefit those at risk who aren’t identified presymptomatically. Incentives to develop rare disease products can increase availability, though extending market exclusivity may limit access.

- FDA should assess the ways by which it incentivizes laboratories to develop and make available new rare disease diagnostic and screening tests.

#### 4.2.2. Costs of Treatments

In the US, all babies are screened regardless of resources, insurance, or other third-party coverage. However, until there is uniform, fair, and established access to essential treatments for conditions diagnosed through NBS, we will have failed to fulfill the obligations we assumed by promoting such screening programs. Many of the financial issues faced by new treatments involve insurance access and coverage policies that vary by State. For example, for many IEMs, dietary therapy is the only effective treatment [73,74,75,76], an important fact that cannot be overstated. Individuals are affected for life. Therefore, coverage for therapies must be lifelong. Although not inherently high-cost, medical foods do increase costs for families. While families have attempted legislative interventions on a State-by-State basis, many policy experts have asserted that a federal measure enacting uniform provisions for these required treatments would provide the best security for families.

To bolster access to follow-up services, ACHDNC, in its capacity as advisory to the Secretary of HHS, should inform and address payer coverage policies by:

- providing expert advice regarding the coverage policies for all NBS-identified patients, recommending coverage policies for the ACA by disposing of the disease-by-disease naming approach in the ACA’s EHB coverage policies, and reframing as coverage of any condition on the NBS RUSP,
- ensuring coverage for all IEM patients requiring medical foods for treatment (criteria for the US to be defined by ACHDNC),
- ensuring coverage of all NBS-identified cases through their transition to adult medicine,
- ensuring that treatment coverage is mandated for all private insurance plans and federal health programs.
  a. In the US, health insurance plans governed by the Employer Retirement Income Security Act (ERISA) should not be exempt,
- assessing programs that seek to redistribute costs for patients and families with rare diseases to society through reinsurance programs.

Additionally, pharmaceutical treatments may be expensive, as evidenced by the $2.1 million per case cost of the first gene therapy. Historically, the argument has been made that rare diseases have a relatively low overall economic impact. However, as the NBS panels of rare treatable conditions grow, the aggregate costs of expensive treatments could become rate-limiting. The ACA has directly covered NBS screening-related costs for a relatively small proportion of the US population while significantly impacting Medicaid expansion and setting a precedent by covering preventive care and NBS. Nevertheless, it’s not uncommon for providers to have to seek preauthorization or to challenge insurer decisions based on medical necessity for service reimbursement from Medicaid. When medical benefits accrue to very small proportions of society, consideration of options for this group is increasingly important, particularly as NBS expands.

ACHDNC, as advisory to the Secretary of HHS, should inform and address access to insurance by ensuring that:

- the preferred intervention for some metabolic diseases, medical foods, are regulated as medical products rather than as dietary supplements to ensure access, quality, and coverage,
  a. Coverage of ‘prescribed’ medical foods should be expanded to Medicare and Medicaid.
- rules established by HHS that could determine the minimum yearly coverage for all health insurance plans don’t pre-empt State standards that may require a higher minimum standard,
- all conditions added to the RUSP are listed with Social Security Disability, and/or compassionate care allowances are available for the treatment of conditions on the NBS RUSP,
- individuals identified by NBS, diagnosed, and found to have an adult-onset form of a disease aren’t subjected to discrimination in health care insurance coverage or pricing when majority age is reached,
- interventions covered include treatments for all conditions recommended to the RUSP by the Committee.

#### 4.2.3. Cost of Quality Control/Assurance/Improvement for NBS Systems

The challenges of maintaining sample repositories that provide the needed positive controls for mandated screens are increasing as the diseases in NBS become rarer, and the intragenic variation grows. Further, the need for samples to validate new technologies and new tests are growing as the number of candidate conditions for NBS expansion grows.

- NBS jurisdictions (done through ACHDNC in the US) should consider the national biospecimen needs (financial and infrastructure) to support the maintenance of quality and technology assessment in State NBS programs.

#### 4.2.4. Ensuring Large Broadly Representative Pilot Studies

The role of federal partners in NBS pilots is outlined in the NBSSLA and mandates federal agencies to fund some of those efforts. NICHD is the lead NIH institute for NBS pilots of candidate conditions. It uses a primary contract with a supplemental funding mechanism to activate a pool of State programs to conduct specified pilot studies. However, system response capacity is limited. Therefore, designing an NBS pilot structure that is interactive, iterative, and synergistic across the system will be important in preparing it for expansion. Identifying decision points along and within the pathway to approval is needed, including when pilot study and nomination data are sufficient. Clinical trials of cleared or approved drugs in infants were rarely included prior to NBS. When included in the course of a pilot study, such trials should be well-coordinated within the pilot study to neither compromise FDA oversight nor the assessment of pilot study results. Rare disease accommodations in regulations must align with the strength of the underlying science when key diagnosis and treatment questions can’t be answered through recurring similar cases.

- To capture the diversity of the population, both nationally and locally, the final critical stage of assessing the appropriateness of adding a new condition to NBS, the population-level clinical validation step, should be coordinated with local Public Health, such as maternal and child health as well as with NBS programs.
- Multiple diverse and populous State NBS programs should be encouraged to participate (and incentivized if necessary) to develop sufficient pilot study data to reliably evaluate the proposed screen in a reasonable amount of time.
- Decision points should be defined on the path from a pilot study to a national recommendation, such as an addition to RUSP, that addresses predictive values, false-positive screening rates, detection rates, or other measures.

#### 4.2.5. Funding Pilot Study Infrastructure

An organized national system and infrastructure that is harmonized with similar systems in other countries is needed that acknowledges the challenges of making screening for rare diseases available while ensuring ongoing development and the data sharing that informs decision-making and improves clinical practice. In the absence of a national healthcare system, centralized registries and databases will be needed along with incentives (e.g., coverage of service delivery fees) for obtaining data while ensuring the privacy of patient information.

NBS systems must balance the need for statistical power during NBS pilot studies against the long time period needed to identify and characterize a sufficient number of rare cases during pilot studies. To accomplish this, synergistic data systems must operate longitudinally and inform several needs in NBS.

Recommendations specific to the United States include that HHS, in consultation with ACHDNC, should:

- study the feasibility of supporting a centralized data warehouse such as the NBSTRN’s longitudinal pediatric data resource (LPDR) that captures pilot study data related to the clinical validity of the use of a particular screening test. Alternatively, access to such data in a federated data system in which data remains locally held, with agreements for sharing particular data fields, will be needed.
- develop rules for databases or registries that support national NBS data sharing while ensuring the protection of patient privacy.

#### 4.2.6. Funding Multisite/Multi-condition Pilot Studies

An additional consideration in successful NBS implementation is the financing of the program from the initial population-level analytical and clinical validation pilot studies through the implementation of an ongoing population-based program while supporting diagnostics and long-term management. As effective treatments for more conditions become available and those conditions become candidates for NBS, the costs of keeping NBS panels current will increase. Traditional condition-by-condition pilot studies of rare diseases are expensive due to the need for a large, unselected, diverse population. Therefore, efficient multidisciplinary pilot studies of multiple conditions are increasingly important. Pilot studies grouping multiple conditions by, for example, technology platforms used, subspecialty provider groups who follow-up screen-positive infants, and disorder groups (e.g., hemoglobinopathies) could be constructed. Risk sharing is an increasingly utilized mechanism for spreading very high short-term investments benefiting small proportions of the population over the long term. In public-private partnerships, financial risks can be shared when government develops a regulatory infrastructure that facilitates rare disease screening and treatment. Successes of this type have generally occurred with therapeutics and may serve as models.

To ensure that NBS programs can actively participate in NBS pilot studies, the ACHDNC, as advisory to the Secretary of HHS, should explicitly recommend the inclusion of NBS funding that includes:

- for final population-level pilot studies that maximize State participation and the resulting diversity of the data collected.
- to implement new conditions as part of federal/State NBS program funding.

When genetic diseases become candidates for NBS expansion, we may know little about caring for the condition as a manageable chronic disease.

- There should be continuity in data collection between the initial clinical validation pilot study used to develop the NBS test and a post-market surveillance period during which additional cases are identified and clinically characterized to inform NBS test performance and clinical care for individuals with these conditions.
- The ACHDNC should work with FDA to outline a process that ensures continuity between pilots and post-market surveillance, including drug approvals for use in newborn populations.

### 4.3. National Quality Assurance Infrastructure

#### 4.3.1. Improving Test Performance

The process of improving NBS test performance begins during the pilot studies. The first-tier test of the NBS screen may or may not be adequate to attain high sensitivity and specificity. Second-tier tests may be added to the NBS algorithm to improve PPV, a measure of clinical validity. Analysis tools for refining cut-offs include machine learning tools such as the Collaborative Laboratory Integrated Reports (CLIR) [77] that can manage normal and abnormal reference ranges and results, including the clinical performance of tests. Although analytical validity can be established on a low number of screened babies, the variable expressivity of genetic diseases requires a larger population to better understand them clinically because analytical targets are often agnostic to the clinical expression of disease. Even after analytical validity is established, there is often a need for more extended clinical validation studies. Analytically, it may become clear that the screening test has an unacceptable recall or false positive rate within certain subpopulations, for example, premature infants in the neonatal intensive care unit (NICU). On the clinical side, the size of the population with late-onset disease or with very mild or nonpenetrant cases may be underestimated and will need to be considered in the context of a screening program and may ultimately make screening infeasible. Complications arise when some aspects of a disease (incidence, age of onset, penetrance, range of severity) are poorly understood before the population pilot study. To capture changes in phenotypes among clinically unaffected cases, longitudinal case-level data and very large numbers of infants may be needed to find the rare cases among the vast unaffected and to account for the clinical variability among those identified with risk factors.

Databases or systems that capture or link to pilot study data and extend it into formal screening are needed. These tools should be capable of capturing longitudinal updates of individual patients’ clinical status to ultimately inform long-term outcomes in those treated.

The rarer the disease, the more disproportionate the impact of false-positive and negative results on test performance, suggesting that a different approach from analytical validation is needed for clinical validation. The size and diversity of the infant population involved in pilot studies must be maximized. International data can inform the population diversity needed. The US should:

- Require and incentivize, as needed, the involvement of multiple States in pilot studies.
- Establish and support tools (e.g., cut-offs, CLIR) that manage normal and abnormal reference ranges and results, including the clinical performance of tests needing comparative analysis.
- Develop standards to address whether a minimum level of PPV should be the goal of NBS development and implementation or if there is an acceptable maximal false-positive screening rate that minimizes impacts on families. Both have to be balanced against the incidence of the condition and its severity in the population, as well as the risks of interventions.

One approach to collecting more robust population data is to introduce conditions into screening on a provisional or conditional basis with ongoing evidence development in centralized data systems (i.e., the equivalent of post-market surveillance for a drug in a manufacturer’s database under the ODA) that would ultimately lead to a final recommendation about formal inclusion (or not) on the RUSP. Programs such as those for the accelerated approval process already include FDA’s ability to require post-market surveillance, which is important when dealing with conditions with considerable clinical variability. A partnership between FDA and NIH addressing this process would be required.

- FDA/HHS should establish rules to:
  a. expand rare disease access to treatments under the ODA, access to rare disease laboratory diagnostic and screening tests should be ensured.
  b. refocus the HDE on clinical and public health laboratory testing and screening for rare diseases rather than on traditional device manufacturers.
- NIH/HHS should expand support of pilot studies on the clinical performance of NBS tests. These pilot studies would fulfill their critical role in accessing the newborn population, thereby providing the range of population diversity needed.
  a. As a requirement for funding, submission of clinical and laboratory data into a database should be required to satisfy the post-market surveillance needs of a two-step NBS process that balances access with continued knowledge development.

Opportunities to improve molecular test result interpretation also exist that can benefit diagnostic and screening uses when used as 2nd tier tests in an NBS algorithm. FDA has recognized the value of innovation in centralized clinical validity databases such as ClinGen’s clinical curation databases [78,79]. In addition to FDA, CMS will have an important role in aligning coverage decisions with the inherent constraints of rare diseases.

- NIH/HHS should prioritize NBS gene and variant curations in ClinGen.
- FDA/HHS should capture NBS test validity data in FDA-recognized clinical validity databases.

#### 4.3.2. Improving the System in which NBS Operates

A critical question involving rare diseases is when research ends and quality improvement begins. This question concerns the differences between research, quality improvement, and standard of care. Under the Common Rule, research requires consent while quality improvement does not. Routine healthcare delivery for rare disease patients is perpetually in a place between the standard of care and research, i.e., clinical investigation that acknowledges the need to practice at the cutting edge of accumulating knowledge to continuously improve disease understanding as new cases emerge.

HHS should clarify the boundaries between research, clinical investigation, and standard of care concerning rare genetic diseases to manage the need for consent when laboratories are implementing an already analytically and clinically validated diagnostic test at the population level.

Standardizing clinical terminologies required to digitally capture patient data during pilot studies is critical to interoperability. Structured case-level data/digitalization is central to interoperable communication. It is unlikely that national electronic clinical data systems will improve this in the short term, which leaves access to long-term follow-up data needed by NBS uncertain. The data collected and information systems used can fulfill two additional needs of NBS if sustained beyond the pilot study: (1). Case-level data collection can continue through pilot studies and into post-market surveillance after initial placement on the RUSP when clinical variability is being characterized and (2). after a condition is added to the RUSP, long-term outcome data is needed to understand that the benefits expected from including a condition in NBS are being realized. A subset of that data could inform a periodic review of conditions in NBS to ensure that the benefits continue to be realized.

HHS should:

- Extend consent to allow case-level data access needed for pilot studies and use in LTFU data collection and outcome analysis.
- Extend this data access need to in-nursery screens where there is considerable State-to-State variability and significant deficiencies in reporting these data back to State programs.

In the absence of EHRs structured to capture case-level data able to support NBS, synergistic systems will need development in order to operate longitudinally and inform the needs of NBS. It will be necessary to extend consent to allow case-level data access for pilot studies and use in LTFU data collection and outcome analysis. It will be important to extend this data access need to in-nursery screens where there is considerable State-to-State variability, and significant deficiencies in reporting data back to State programs exist.

ACHDNC should assemble expertise to advise on requirements for building a system that interoperably captures data during pilot studies that can be extended into post-market data collection and to periodic reviews of ongoing NBS test performance.

Evolving knowledge of genetic diseases has brought new considerations for NBS bioethics research that can be integrated into NBS pilot studies and practice [41,80]. Informed consent requirements vary across the States. Some use an opt-out consent, others a formal opt-in; some States choose to waive opt-in consent with an opt-out option under the Common Rule. Obtaining meaningful informed consent at all birthing sites across the country is unmanageable, particularly given the statistical demands of evaluating rare disease needs at a population level.

HHS should recognize State preferences for waivers and opt-out consent processes by supporting high-quality decision-making information appropriate to multiple levels of education rather than for opt-in consent that neither birthing center staff nor their resources can support.

As more genetic conditions are found to have mixed ages of onset or increased levels of nonpenetrance, ACHDNC should assess the risks (e.g., access and costs of insurance at majority age) for cases identified by NBS.

### 4.4. Data and IT Communications Infrastructure

#### 4.4.1. IT, Informatics, Communication

Due to the medical emergency status of infants who screen positive for some conditions in NBS, systems to rapidly move information between NBS programs, local providers, and patients/families are required. The current COVID-19 pandemic has highlighted significant challenges to the timely and efficient distribution of public health information. Further, a systematic collection of data on race and ethnicity that enables tracking of disparities and equity is needed. Countries with centralized healthcare systems have advantages in accessing data tied to healthcare delivery and public health, allowing for a “real-time” decision-making process. NBS systems can improve linkages between birthing hospitals and public health programs that can provide timely follow-up for screen-positive newborns and assure access to the treatments that allow improved outcomes. The obvious collaborator in developing hospital standards is the Joint Commission which sets standards for hospitals in tracking babies with “critical results”. Their standards and resulting data have been invaluable for similarly rare cancers.

The ACHDNC should work with the Joint Commission to consider the standards that hospitals must meet to ensure safe and effective NBS test delivery, including the tracking of positive infants with critical results, even in cases where results of hospital NBS are returned for follow-up after a newborn has been discharged.

- The ACHDNC should undertake a comprehensive assessment of current interoperability issues in NBS. Many children’s programs such as lead screening, Early Periodic Screening Diagnosis and Treatment (EPSDT), and HL screening remain disconnected from the NBS Programs such that better integration would improve healthcare for children.
  a. Public health and care providers should improve their interoperability capacity to share the diagnostic and outcome data needed to improve NBS delivery.
  b. Investments in public health information systems to improve the interoperability of data sharing related to infectious diseases should consider the needs of NBS programs in system design.

#### 4.4.2. Government Public Policy and Public Health Role

NBS programs and staff have been subjected to unprecedented work and personal challenges during the COVID-19 pandemic leading to significant staff turnover and the loss of some public health authority. However, appreciation of the struggles of public health systems during the COVID-19 pandemic has created the best chance for change in many years.

Two public health programs in the US impact most newborns: NBS and childhood immunization programs. The law that describes the National Vaccine Program includes central coordination, with its own budget and staff, and an Interagency Vaccine Group. Although the ACHDNC has major responsibilities concerning NBS in the US, there remains no clear administrative structure that organizes the intersection between the federal and State public health programs or that organizes the many federal agencies involved in public health—specifically NBS and genetics/genomics. Thus far, the intersection with State public health and NBS programs is accomplished through directed funding from various federal agencies.

However, entities charged with facilitating inter-organizational coordination have the potential to be powerful and effective under certain conditions. Notably, a balance of authority and resources is needed to enable such entities to be effective in most jurisdictions. A clear delineation of roles and responsibilities is critical for effective collective action. In addition, organizational mechanisms, as within HHS in the US, can direct resources to collective activities rather than individual organizational goals. In a rapidly developing area that is likely to bridge research involving new technologies and knowledge, rare diseases, and population health, a centralized coordinating effort is important.

- An office on newborn and genomic screening that provides strategic leadership and management while encouraging collaboration, coordination, and innovation among federal agencies and stakeholders to reduce the burden of genetic diseases is needed. In the US, HHS should create such an office within the Office of the Assistant Secretary of Health.
- The ACHDNC should recommend to HHS that they provide a transparent process of the decision-making and recommendation process of the Interagency Coordinating Council (ICC).
- The ACHDNC, in collaboration with public health and maternal and child health programs, should convene an intergovernmental panel to address workforce and financial shortages and IT needs.
- The ACHDNC should recommend to HHS a clearer process of interagency coordination through the ICC, including which HHS agencies should serve on the ICC.

## 5. Conclusions

The rapid acquisition of knowledge about the etiology of thousands of genetic diseases and the resulting development of treatments for these rare diseases has presented more candidate conditions for NBS. In addition, the rapid developments in genome sequencing, for which costs have now decreased to a price equivalent to many other complex medical tests, increase the possibilities of their clinical application, including in the NBS processes. However, the current systems for moving that new knowledge into NBS are inadequate or undeveloped. This disconnection between research and development programs is particularly apparent in population-level screening for rare diseases or the even rarer sub-targets of a disease, especially when NBS is desired as a strategy for presymptomatic identification. Developing an approach in which the significant benefits of identifying and treating at-risk infants are maximized is critical. What remains to be completed is a systems-based approach that builds confidence in population screening of specific conditions and their diagnoses and treatments.

Although many of our recommendations are specific to US systems, the underlying issues are often shared among international NBS programs that can adapt them as appropriate. Recommended changes include: (1) more specific delineation of the conditions being screened and the reasons why and when screening occurs; (2) a pilot study system with financing that includes the ability of NBS programs to participate; (3) maximizing the utility of data acquired in pilot studies by ensuring that it is continually collected throughout implementation and into the yet-to-be-defined outcome data needed to periodically evaluate screening test performance; (4) managing costs of new technologies and other infrastructure from population pilot studies through a post-market surveillance phase in which the diagnosis and management data of these rare and often-variable NBS conditions can be aggregated to improve newborn care; and (5) focusing on the interoperability needs of NBS programs across all stakeholders.

These recommendations necessitate coordination of the research agenda for condition evaluation and approval/disapproval for NBS, treatment(s)/management, and candidate-condition pilots with ACHDNC and various federal agencies. This systems-based approach still requires the establishment and use of centralized or federated institutional review boards and enlisting patient advocacy groups into patient-centered research models. Ultimately building such a system will enhance collaborative opportunities, distribute costs, and share resources across the public health and health care delivery systems as well as the rare disease research communities while prioritizing the improvement of care for those identified in NBS.

## Figures and Tables

**Table 1:** Roles of US federal agencies in NBS.

Federal Agencies		
CDC		**Support national newborn screening program for quality assurance.** Also provides guidance and oversight for the control of infection and chronic illness; preparedness for new health threats.
NIH		Support for research and development of new public health approaches, therapies, and treatments. **Relevant research programs include Rare Disease and Genetics/Genomics**
FDA		Responsible for protecting public health by assuring the safety, efficacy, and security of human and veterinary drugs, biological products, medical devices, our nation’s food supply, cosmetics, and products that emit radiation. **Relevant programs include Orphan Drug Program**
HRSA		**Supports the only federal Genetic Services Program, including the ACHDNC**. Supports programs for health and public health infrastructure, training of health professionals and distributing them to areas where they are needed most, providing financial support to health care providers, and advancing telehealth. HRSA programs provide equitable health care to people who are geographically isolated and economically or medically vulnerable. This includes programs that deliver health services to people with HIV, pregnant people, mothers and their families, those with low incomes, and residents of rural areas.
CMS		Serves Medicaid and Medicare beneficiaries
**State Agencies**		
Of 50 state public health agencies, 29 are independent agencies, and 21 are a unit of a larger umbrella agency; 27 have a State board of health or similar entity.		**Newborn screening** Programs and policies to address maternal–child health, environmental health, chronic illness, tobacco control, and infectious disease Public health emergency response Vital statistics Infectious and chronic disease surveillance Maintenance of immunization registries Licensing and regulation of health care service providers Laboratory testing, including foodborne illness testing and influenza typing

**Table 2:** Timing of NBS Expansions.

Years	Conditions in NBS
1960s	PKU
1970s	Sickle cell (SS) disease (SCD) and other S allele conditions, congenital hypothyroidism (CH),
1980s	Galactosemia (GAL), maple syrup urine disease (MSUD), congenital adrenal hyperplasia (CAH), biotinidase def. (BIO)
1990s	No uniform approach to screened conditions
2000s	Cystic fibrosis (CF); Medium-chain acyl CoA Dehydrogenase deficiency (MCAD); Very Long-chain acyl CoA Dehydrogenase deficiency (VLCAD); Long-chain acyl CoA Dehydrogenase deficiency (LCHAD); Trifunctional Protein deficiency (TFP); Carnitine uptake/transport; Methylmalonic aciduria (MMA) (mutase); MMA (cobalamin); Propionic Acidemia (PA); isovaleric acidemia (IVA); 3-methyl crotonyl carboxylase deficiency (3MCC);3-hydroxy 3-methylglutaryl-CoA lyase deficiency (3H3MG); Holocarboxylase def.; Beta-keto-thiolase deficiency (BKT); Glutaric acidemia (GA 1); ASA; Citrullinemia Type 1 (CIT 1); Homocystinuria (HCU); Tyrosinemia type 1 (TYR) 1; Severe Combined Immunodeficiency (SCID); hearing loss (HL)
2010s	Spinal Muscular Atrophy (SMA); Pompe; Mucopolysaccharidosis I; Critical Cyanotic Congenital Heart Disease (CCHD); X-linked adrenoleukodystrophy (X-ALD)
2020–2021	None added to RUSP

**Table 3:** Federal agencies supporting NBS research.

Group	Funding Agency	Attributes	Focus
National Cancer Cooperative Study Groups	NCI	National coverage Integrated laboratory and clinical services Participation of regulators and payers	Rare cancers Research component Rapid clinical translation
Clinical Translational Science Award Program (CTSA)	NIH/NCATS	More than 50 US medical research institutions National clinical trials network Rare disease subgroups	Workforce training in translational science Clinically affected individuals Informatics
Newborn Screening Translational Research Network (NBSTRN)	NICHD/NIH	Research infrastructure Standardized data dictionaries	NBS research infrastructure Standardized data dictionaries NBS pilot studies
Newborn Sequencing in Genomic Medicine and Public Health (NSIGHT)	NHGRI, NICHD/NIH	Partnerships with NBS programs ELSI component	Comparative assessment of new technology with traditional NBS methods ELSI issues
Rare Disease Clinical Research Network (RDCRN)	NCATS/NIH	Multi-institutional networks of investigators	Rare diseases

## Supplementary Materials

The following supporting information can be downloaded at: https://www.mdpi.com/article/10.3390/ijns8030041/s1.

## Abbreviations

AAP	American Academy of Pediatrics
AAV	Adeno-associated virus
ACA	Affordable Care Act of 2010
ACHDNC	Advisory Committee on Hereditary Diseases in Newborns and Children (HHS)
ACMG	American College of Medical Genetics
ASO	Antisense Oligonucleotide
BIO	Biotinidase deficiency
CDC	Centers for Disease Control and Prevention (HHS)
cfDNA	Cell-free DNA
ClinGen	The Clinical Genome Resource
CMV	Cytomegalovirus
CLIA	Clinical Laboratory Improvement Amendments
CLIAC	Clinical Laboratory Improvement Advisory Committee
CLIR	Collaborative Laboratory Integrated Reports
CORN	Council of Regional Networks for Genetic Services
CRISPR	Clustered regularly interspaced short palindromic repeats
CRISPR-Cas 9	CRISPR-associated protein 9
DMD	Duchenne muscular dystrophy
EHR	Electronic health record
EPSDT	Early Periodic Screening Diagnosis and Treatment
eRNA	Endless Ribonucleic Acid
ES	Exome sequencing
ExAc	Exome Aggregation Database
FDA	Food and Drug Administration (HHS)
GAL	Galactosemia
GAMT	Guanidinoacetate methyltransferase
GAO	Government Accountability Office
gnomAD	Genome aggregation database
GS	Genome sequencing
HDE	Humanitarian Device Exemption
HHS	US Department of Health and Human Services
HL	Hearing loss
HRSA	Health Resources and Services Administration (HHS)
HCY	Homocystinuria
ICC	Interagency Coordinating Council
IEM	Inborn errors of metabolism
IND	Investigational new drug
IT	Information technology
IOM	Institute of Medicine
LPDR	Longitudinal pediatric data resource
LSD	Lysosomal storage disorder
LTFU	Long-term follow-up
MSUD	Maple Syrup Urine Disease
MIM	Mendelian Inheritance in Man
MPS II	mucopolysaccharidosis type II
mRNA	Messenger Ribonucleic Acid
MS/MS	Tandem mass spectrometry
NBS	Newborn screening
NBSSLA	Newborn Screening Saves Lives Act of 2008
NBSTRN	Newborn Screening Translational Research Network
NCATS	National Center for Advancing Translational Research
NHGRI	National Human Genome Research Institute (NIH)
NICHD	National Institute of Child Health and Human Development (NIH)
NICU	Neonatal intensive care unit
NIH	National Institutes of Health (HHS)
NORD	National Organization for Rare Diseases
NRC/NAS	National Research Council of the National Academy of Sciences
NSIGHT	Newborn Sequencing in Genomic Medicine and Public Health
NSQAP	Newborn Screening Quality Assurance Program (CDC)
ODA	Orphan Drug Act of 1982/83
OMIM	Online Mendelian Inheritance in Man
ORD	Office of Rare Diseases (NIH)
PKU	Phenylketonuria
PPV	Positive predictive value
RDCRN	Rare Disease Clinical Research Network
RUSP	Recommended Uniform Screening Panel (US.)
SCD	Sickle cell disease
SCID	Severe combined immunodeficiency
SF	Secondary findings
siRNA	Small interfering Ribonucleic Acid
SMA	Spinal muscular atrophy
STFU	Short-term follow-up
TREC	T-cell Receptor Excision Circles
VUS	Variant of uncertain significance
X-ALD	X-linked adrenal leukodystrophy
