# Supplementary material for: The Progress and Future of US Newborn Screening
Source: Int J Neonatal Screen. 2022 Jul 18;8(3):41. doi: 10.3390/ijns8030041 (PMC9326622; doi:10.3390/ijns8030041)
Supplement: Supplementary file 1 [file IJNS-08-00041-s001.zip › IJNS-1649552-supplementary.pdf]

**Newborn Screening: History and Advancement of Newborn Science and Technology**
Michael S. Watson, MS, PhD, Michele A. Lloyd-Puryear, MD, PhD, R. Rodney Howell, MD

**1. Introduction**

The history of newborn screening (NBS) has evolved as disruptive scientific and technological advances have changed the potential of NBS to improve population health. It has been 60 years since NBS was formally initiated in the U.S. as a state public health program to screen newborns for phenylketonuria (PKU). Prominent among NBS policy and scientific accomplishments (Tables S1a and b) are the recognition of the development of principles upon which to base NBS actions and the need for: 1) a national scientific decision-making process; 2) the development of national and state-based quality assurance systems; 3) the development of comprehensive national information systems and program standardization; and 4) development of NBS information systems; and 5) development of national policy for NBS program regulation and standardization. We consider here the past and current drivers of development of NBS programs.

**Table S1.a.** Timeline of major developments in newborn Screening (1960 – 2022): Science and Technology.

**SCIENCE and TECHNOLOGY**

- 1900, Garrod shows alkaptonuria transmits in a typical Mendelian recessive manner.
- 1900, Galactosemia, an inborn error of galactose metabolism, was first described by von Ruess
- 1934, Følling discovers phenylketonuria (PKU)
- 1949, Pauling studies molecular biology of sickle cell anemia
- 1953, Følling develops test for detecting PKU
- 1953, Bickel determines dietary treatment for PKU
- 1953, Watson and Crick elucidate structure of DNA molecule
- 1954, Maple syrup urine disease (MSUD) was first described in 1954 by Menkes et al. as a progressive neurologic degenerative disorder.
- 1960, Dancis et al. established that the metabolic block in MSUD is at the decarboxylation of branched-chain alpha-ketoacids derived from leucine, isoleucine, and valine.
- 1961, Guthrie creates first NBS test for PKU
- 1963, Galactosemia (GAL) was the second disorder found to be detectable by NBS with methods developed by Robert Guthrie and Ken Paigen.
- 1965, Thirty-two American states had enacted screening laws, all but 5 making the PKU NBS compulsory
- 1968, New York starts pilot testing newborn screening for GAL and MSUD
- 1968, Wilson and Jungner principals published.
- 1970, Forty-five states had enacted NBS laws
- 1973, Screening methods for CH and SCD developed
- 1990, MS/MS applied to NBS
- 2010, All states are screening for more than 30 conditions (many by MS/MS) in NBS
- 2012, CRISPR/Cas 9 gene editing systems discovered
- 2017, NSIGHT program demonstrates roles for genome sequencing in NBS
- 2018, First gene therapy for an NBS condition cleared by FDA: Zolgensma® for SMA
- 2019, New York ScreenPlus pilot study program funded by NICHD
- 2021, More than one hundred gene targeted therapies reported by FDA to be in late-stage clinical trials.

**Table S1.b:** Timeline of major developments in newborn Screening (1960 – 2022): Legislation, Regulation and Policy

**LEGISLATION, REGULATION, AND POLICY**

- 1961, NICHD created.
- 1962, Children's Bureau of the Department of Health, Education, and Welfare and state departments of public health promoted mandatory NBS. Funded pilots/research for PKU screening.
- 1972, National Sickle Cell Disease Control Act establishes SCD research centers and clinics.
- 1975, review of genetic screening and NBS by National Research Council of National Academy of Science (NRC/NAS).
- 1976, Genetic Diseases Act was authorized to fund NIH and HRSA to establish national programs for basic and applied research and training and programs for testing, counseling, information, and education programs with respect to genetic diseases.
- 1976, Medical Devices Act.
- 1978, NSQAP created at CDC [recommendation from NRC/NAS report].
- 1978, Genetic Services program created at MCHB/HRSA.
- 1983, FDA Office of Orphan Products Development was created through the Orphan Drug Act of 1983 to provide incentives to those developing drugs for rare disorders.
- 1982, National Organization for Rare Diseases (NORD) established.
- 1983, Council of Regional Genetics Networks (CORN) established.
- 1987, NIH and HRSA convened a consensus development conference on *Newborn Screening for Sickle Cell Disease and Other Hemoglobinopathies.*
- 1987, International Society for Neonatal Screening established.
- 1989, National Human Genome Research Institute established to map human genome.
- 1993, NIH Task Force on Genetic Testing was formed. Its report in 1995 addressed the many intended uses of a genetic test from diagnosis and family genetics through population uses such as carrier screening and NBS.
- 1997, CLIAC addressed oversight under CLIA '88 of the rapidly growing area of genetic testing.
- 1998, American Academy of Pediatrics (AAP) NBS Task Force formed. Report published 2000.
- 2002, Children's Health Act. Establishes The Secretary's Advisory Committee on Heritable Disorders in Newborns and Children (ACHDNC) and the Heritable Disorders Program.
- 2002, Rare Diseases Act of 2002 established the Office of Rare Disease at NIH to recommend a research agenda and to coordinate related activities.
- 2002, American College of Medical Genetics (ACMG) NBS Expert Group established.
- 2003, NIH establishes the Rare Disease Clinical Research Centers.
- 2003, ACHDNC holds inaugural meeting.
- 2004-2005, ACHDNC reviews ACMG report and approves in 2005. The recommended conditions became the basis of the ACHDNC's first RUSP.
- 2008, Newborn Screening Saves Lives Act (NBSSLA) was signed into law.
- 2009, NIH/NICHD Hunter Kelly NBS Research Program established at NIH by NBSSLA.
- 2015, NBSSLA reauthorized with new consent requirement for 'research' studies.
- 2015, NewSteps replaces NNSGRC as national data center for NBS.
- 2018, NBSTRN publishes recommendations for inclusion of ELSI in NBS.
- 2022, Reauthorization of NBSSLA delayed.

**2. Newborn Screening: 1930s – 1990s**

NBS has been driven since its beginning in the early 1960s by rapid developments in science and technology, government policies, workforce development, and consumer advocacy. The scientific groundwork for NBS began with discoveries in the 1930s through the 1960s including a treatment for PKU discovered in 1953 by Horst Bickel [1,2] followed by the critical development of a bacterial inhibition assay for the detection of PKU using blood absorbed onto special collection paper by Robert Guthrie.[3-5] The incorporation of a manual hole-puncher to obtain discs from blood spots on filter paper (used in the newborn period by Guthrie) replaced an unreliable low-throughput diaper urine screen[6] with a high-throughput centralized laboratory blood test that made a state public health program role in NBS feasible. Funded by the federal Children's Bureau, hospitals in Massachusetts initiated the first demonstration project of NBS for PKU NBS in 1962. [5]. Interestingly, Delaware is reported to have included NBS regulations in 1962 using a broad interpretation of existing statutes [7]. By 1970, 45 states required screening for PKU [8]. The first regional programs were organized concurrently in the 1960s by Massachusetts (among New England States) and Oregon (among Northwest states) to centralize their laboratory screening. Successful implementation of PKU NBS led to additional bacterial inhibition assays for screening of other rare metabolic conditions, some less effectively treated than PKU and others such as histidinemia subsequently determined to be benign. Specimen preparation was laborious and additional screening tests were not readily added to screening panels. Automated punching opened the way for expanded screening panels of up to four disorders that could be simultaneously punched/prepared for analysis [9]. By 1975, Dussault had developed NBS for congenital hypothyroidism using radioimmunoassay (RIA) screening for thyroxine [10,11]. The pace of addition of conditions to NBS continued slowly into the 1990s usually involving initiation of a singular screening test for a single disorder (Table S2).

**Table S2:** Conditions in NBS (1960 – 2000).

| Years | Conditions in NBS |
|---|---|
| 1960s | PKU |
| 1970s | Sickle cell (SS) disease (SCD), other S-allele associated conditions,<br>Congenital hypothyroidism (CH) |
| 1980s | Galactosemia (GAL),<br>maple syrup urine disease (MSUD)<br>congenital adrenal hyperplasia (CAH)<br>Biotinidase deficiency (BIO) |
| 1990s | No uniform approach to screened conditions |

Medical genetics was in its infancy in the 1950s. Watson and Crick modeled the DNA double helix and chromosomal disorders, and genetic metabolic conditions were being identified. The true number of chromosomes was determined to be 46 in 1956 rather than the 48 that had been claimed until then. The 1st demonstration of a DNA-based diagnostic test was reported for the prenatal detection of the sickle cell S-allele [12,13]. The resolution of gene mapping was improving in the 1970s with the advent of somatic cell hybridization that allowed specific traits to be assigned to specific chromosomes. Fields like biochemistry, genetics and genomics that were likely to integrate with NBS also were quickly developing, having led to rapid elucidation of the underlying abnormalities characteristic of specific genetic diseases.

The 1st edition of McKusick's Mendelian Inheritance in Man (MIM), published in 1966 included 1486 entries derived from the biomedical literature, most of which were 'uncommon' phenotypes. Defining by their patterns of inheritance with somatic cell hybridization allowed traits to be assigned to chromosomes. Over the next 50 years, specific pathogenic variants in these genes and associated phenotypes were identified, enabling genetic testing and screening [14].

Fields such as biochemistry, genetics and genomics that were likely to integrate with NBS also were quickly developing, leading to rapid elucidation of the underlying abnormalities characteristic of specific genetic diseases.

Early in the 1990s, improvements were made in automated analysis, in part enabled by the introduction of electrospray ionization, sample-introduction techniques, method validation, and development of automated interpretation systems. Concurrently, the number of specimens analyzed annually increased substantially. During the 1990s, tandem mass spectrometry (MS/MS) was shown to be an effective screening platform in North Carolina enabling simultaneous NBS for many amino acid, fatty acid oxidation, or organic acid disorders and ultimately driving interstate variability in screening panels [15-20].

**2.1.** *Information Technology, Informatics, and Communication*

Information technology (IT) communication tools were just being introduced in the 1980s when the 1st fax machines and microcomputers became available, leading to improved communications and result delivery between the NBS program and the health care delivery system (Table S1a,b). The increasing pace of expansion of NBS tests through the 1970s and '80s along with apparent variation in clinical outcomes of those identified by NBS and subsequently treated, pointed to the need for national data collection, program harmonization/standardization and NBS information systems development, including systems interoperability through IT.

**2.2.** *Governmental Public Policy and Public Health Role*

Significant to NBS, the roles of government and families were critical to the development of NBS programs and support. Eunice Kennedy Shriver, a longtime advocate for children's health and disability issues, became Executive Vice President of the Joseph P. Kennedy Jr. Foundation in 1957 and championed the creation of the President's Panel on Mental Retardation in 1961 and the National Institute of Child Health and Human Development (NICHD) in 1962, as a part of the National Institutes of Health (NIH). In 1962, the Children's Bureau of the federal Department of Health, Education, and Welfare funded pilots/research for PKU NBS.[21] By 1965, thirty-five American states had enacted screening laws.

The increasing pace of expansion of NBS through the 1970s and 80s along with apparent variation in outcomes of those identified with disease through NBS and subsequently treated, pointed to the need for national policy data, program harmonization/standardization and NBS systems improvement. The National Research Council of the National Academy of Sciences (NRC/NAS) [8] was convened in 1975 to develop its first recommendations on genetic testing, including aspects of NBS, thus increasing the role of government in policymaking. Wilson and Jungner in 1968 [22] proposed criteria for screening adults for chronic disease noting:

> *"The central idea of early disease detection and treatment is essentially simple. However, the path to its successful achievement (on the one hand; bringing to treatment those with previously undetected disease; and, on the other, avoiding harm to those persons not in need of treatment) is far from simple though sometimes it may appear deceptively easy.".*

The NRC/NAS reviewed those principles but brought a specific focus to NBS and rare diseases. The NRC report was prophetic in making the following statement,

> *"As new screening tests are devised, they should be carefully reviewed. If the experimental rate of discovery of new genetic characteristics means an accelerating rate of appearance of new screening tests, now is the time to develop the medical and social apparatus to accommodate what later on may otherwise turn out to be unmanageable growth".*

Since then, additional recommendations have been put forth by several professional groups and federal agencies. In 1987, the NIH and HRSA convened a consensus development conference on *Newborn Screening for Sickle Cell Disease and Other Hemoglobinopathies.* The conference concluded that universal screening for SCD should be provided [23]. Other recommendations for NBS came from American Academy of Pediatrics (AAP) in 1999 [24[, the Institute of Medicine (IOM) with Assessing Genetic Risks [25], and the American College of Medical Genetics (ACMG) in 2005 [26]. Except for the reports from the NAS, these review efforts were prompted by federal funding from HRSA/HHS. The AAP report served as a blueprint for many of HRSA's activities through this decade. Indeed, the various commissioned groups' recommendations have been implemented over time, and now there are federal agencies in existence, responsive and responsible to carry out the programs and support research on various aspects of genetics and NBS, including implementation of a federal law that protects consumers from discrimination by their employers and the insurance industry on the basis of genetic information (Table S1a and b).

Regulatory processes also were addressed during the decade (Table S1a and b) and important for NBS and rare diseases, the U.S. Food and Drug Administration (FDA) Office of Orphan Products Development was created through the Orphan Drug Act (ODA) of 1982/83 to provide tax incentives and market exclusivity to those developing drugs for rare disorders. By 1990, Congress had added FDA Humanitarian Device Exemption (HDE) to the Safe Medical Devices Act of 1990 for products intended for diseases or conditions affecting rare disease populations. However, because the genetic testing industry was characterized by laboratory developed tests (LDTs) with low insurance reimbursement there was little impetus to develop and make available FDA-cleared tests and testing devices. As such, the HDE was much less successful than the ODA in developing incentives for industry to innovate in this area.

## 3. Newborn Screening: 2000 - 2009

### *3.1. Scientific Knowledge and Technology Expansion*

The decade began with significant NBS expansion often secondary to the incorporation of tandem mass spectrometry (MS/MS) into NBS programs. Many programs added up to eighteen conditions using multiplex-screening by MS/MS between 1990 and 2010 while CF, SCID, and hearing loss (HL) were added using individual test for each (see Table S2) [27]. In the interest of bringing greater national uniformity to NBS in 2003, HRSA/HHS engaged ACMG to establish the criteria by which conditions are assessed and apply it to the evidence available on 84 genetic conditions and phenotypes already being screened somewhere in the world. Evidence-based medicine standards were just developing but most case-level evidence and data remained inaccessibly siloed in clinics and physician's offices. There were national and international gaps in individual and population-level data on rare disease cases globally. Data on penetrance and the range of disease severity were lacking, hampering decisions on which conditions to include in required NBS.

**Table S3.** Conditions in NBS (2000-2010).

| **Years** | **Conditions in NBS** |
|---|---|
| 2000s | Cystic fibrosis (CF)<br>Medium-chain acyl CoA Dehydrogenase deficiency (MCAD)<br>Very Long-chain acyl CoA Dehydrogenase deficiency (VLCAD)<br>Long-chain acyl CoA Dehydrogenase deficiency (LCHAD)<br>Trifunctional Protein deficiency (TFP)<br>Carnitine uptake/transport (CUD)<br>Methylmalonic aciduria (MMA) (mutase)<br>MMA (cobalamin)<br>Propionic Acidemia (PA)<br>isovaleric acidemia (IVA)<br>3-methyl crotonyl carboxylase deficiency (3MCC)<br>3-hydroxy 3-methylglutaryl-CoA lyase deficiency (3H3MG)<br>Holocarboxylase def.; Beta-keto-thiolase deficiency (BKT)<br>Glutaric acidemia (GA 1)<br>Argininosuccinate synthetase def(ASA)<br>Citrullinemia Type 1 (CIT 1)<br>Homocystinuria (HCU)<br>Tyrosinemia type 1 (TYR) 1<br>Severe Combined Immunodeficiency (SCID);<br>Hearing loss |

Prior to this decade, pilot studies of candidate conditions were largely single-state efforts limited to a few programs with research missions and funding added to their service responsibilities. The statistical demands and costs of running sufficiently large prospective pilot studies for new and rare often-complex conditions under consideration for possible addition to NBS, particularly one at a time, becomes obvious. In 2008, NICHD/NIH contracted with ACMG to establish and operate the Newborn Screening Translational Research Network (NBSTRN) as a bridge between research and clinical investigation to enhance the knowledge base and clinical care and to develop the tools to support large multistate NBS pilot studies.

Population screening increases understanding of the disease and its spectrum of severity. One of the more robust NBSTRN pilot studies evaluated screening for a condition was that done by NBSTRN for severe combined immunodeficiency (SCID) disorders. This very large pilot study highlighted the difficulty in obtaining sufficient statistical power with rare disease studies, even with seemingly very large populations [28]. Central to the solution is the need for latitude in the statistical power requirements when screening for rare diseases. In SCID the number of unique genetic conditions meeting the SCID definition expanded from 25 to 50, highlighting the ongoing rapid accumulation of knowledge on genetic diseases. Importantly for SCID, the screening test, T-cell Receptor Excision Circles (TRECs) [29], brought molecular testing to the first tier of screening algorithms while industry was developing multiplexed gene panel tests for the diagnosis of the many genetic etiologies of SCID. NBSTRN became active in collaborating with state NBS programs and investigators on population-stage pilot studies of Pompe disease, mucopolysaccharidosis type II (MPS II), spinal muscular atrophy (SMA), and X-linked adrenal leukodystrophy (X-ALD), and Duchenne muscular dystrophy (DMD) [30-35].

The NBSTRN pilots also pointed to the need for improved data collection infrastructure. NBS historically includes short-term follow-up (STFU) and long-term follow-up (LTFU). STFU includes the

establishment of a diagnosis, or not, and the plans for or the initiation of treatment. LTFU includes the initiation of and response to treatment, connection to related services, and clinical outcome evaluations to assist in system quality improvements. The clinical outcomes are particularly important in establishing clinical validity and utility. At this time, data from all US NBS programs were voluntarily reported to the Council of Regional Networks and later, the National Newborn and Genetics Resource Center (CORN)/NNSGRC) as a means of self- and inter-program evaluation for quality improvement and public review as a means of program transparency [36]. Ongoing data collection and analyses are essential in supporting the continuation of screening, diagnoses, and treatment and in understanding whether the expected outcomes of screening have been realized.

Data from STFU and LTFU of screen positive newborns has highlighted how biased our views of genetic diseases may be when cases are ascertained through studies of those clinically affected and their families. Unbiased ascertainment typically results in a better understanding of disease incidence, identification of a broader range of disease severity, particularly at the mild end of the disease spectrum. Full population screening, as in NBS pilots, identifies later-onset forms of a disease and provide a less biased estimate of disease penetrance.

### 3.2. *Governmental Public Policy and Public Health Role*

During this 2000-2009 decade there was no clear administrative structure intersecting federal and state public health programs, specifically NBS and genetics (Table S4). A government-wide assessment by the Government Accountability Office (GAO) also pointed to the lack of federal and state coordination [37]. Further, there was no clear administrative structure that organized the many federal agencies involved in public health or specifically, NBS and genetics [25,38].

**Table S4.** Roles of U.S. federal agencies in NBS

| | | |
|---|---|---|
| **Federal** | | |
| | CDC | **Support national newborn screening program for quality assurance.** Also provides guidance and oversight for the control of infection and chronic illness; preparedness for new health threats. |
| | NIH | Support for research and development of new public health approaches and therapies, and treatments. **Relevant research programs include Rare Disease and Genetics/Genomics.** |
| | FDA | Responsible for protecting the public health by assuring the safety, efficacy and security of human and veterinary drugs, biological products, medical devices, our nation's food supply, cosmetics, and products that emit radiation. **Relevant programs include Orphan Drug Program.** |
| | HRSA | **Supports the only federal Genetic Services Program, including the ACHDNC**. Supports programs for health and public health infrastructure, training of health professionals and distributing them to areas where they are needed most, providing financial support to health care providers, and advancing telehealth. HRSA programs provide equitable health care to people who are geographically isolated and economically or medically vulnerable. This includes programs that deliver health services to people with HIV, pregnant people, mothers, and their families, those with low incomes, and residents of rural areas. |
| | CMS | Serves Medicaid and Medicare beneficiaries |
| **State** | | |
| | Of 50 state public health | **Newborn screening** |

| | | |
|---|---|---|
| | agencies, 29 are independent agencies and 21 are a unit of a larger umbrella agency; 27 have a state board of health or similar entity. | Programs and policies to address maternal–child health, environmental health, chronic illness, tobacco control, and infectious disease<br>Public health emergency response<br>Vital statistics<br>Infectious and chronic disease surveillance<br>Maintenance of immunization registries<br>Licensing and regulation of health care service providers<br>Laboratory testing, including foodborne illness testing and influenza typing |

Because of individual champions within federal and state government agencies and professional and consumer advocacy organizations, 2000-2009 marked a decade when federal interest and public support and activities for NBS programs grew greatly, much driven by the Child Health Act of 2000, the Newborn Screening Saves Lives Act of 2008 (NBSSLA) and the creation of the Secretary's Advisory Committee on Heritable Disorders in Newborns and Children (ACHDNC) which formalized acceptance of the ACMG's recommendations for the first national Recommended Uniform Screening Panel (RUSP) [26,27]. As per its legislation, ACHDNC established the criteria by which it would accept nominations and review conditions addition to the RUSP [39]. Unfortunately, while pilot studies to establish whether a condition should be added to NBS panels were numerous, most pilots lacked clear endpoints, such as how many cases needed to be identified. In addition, while rapidly developing IT and informatics tools enabled broad collaborative pilot studies and data sharing, funding was inadequate for a comprehensive and coordinated national system. The implementation of the NBSSLA increased support for funding pilots and NBS public health and services infrastructure, thereby facilitating the incorporation of the RUSP into state NBS panels.

Further government support came with the enactment of the Rare Diseases Act of 2002 which established the Office of Rare Disease (ORD) at NIH to recommend a NBS research agenda and to coordinate related activities. By 2003, NIH had established a rare disease research agenda and to coordinate related activities. By 2003, NIH had established the Rare Diseases Clinical Research Centers (RDCRCs) to fill a significant gap in knowledge related to rare genetic diseases.

Movement towards a national electronic health information system in the U.S. had resulted in tools useful for administrative tasks such as billing but were limited in their ability to manage longitudinal case-level clinical information. More than one-third of local health departments reported their inability to access an electronic surveillance system with data from local emergency departments, which could facilitate early identification of screen positive infants identified through NBS [38,40,41]. In 2007 the American Health Information Community (AHIC) was charged with establishing the standards needed to move towards a) interoperability, b) the use of the most advanced health IT and, c) electronic exchange of health information. Movement of IT forward for NBS occurred when AHIC accepted NBS as a use case to address gaps between public health and the health care delivery system [42]. It resulted in the creation of the *Newborn Screening Coding and Terminology* Guide [43] by the National Library of Medicine (NLM) to promote and facilitate use of health data standards in recording electronic NBS results and interoperability transmitting those results.

**4. Newborn Screening: 2010 – 2019**

By 2010, inclusion of a condition in NBS had become a sign that the disease was understood and could be effectively treated. There was growing consumer involvement as increasing numbers of disease support groups made the addition of a specific condition(s) to the RUSP a main goal of their advocacy. The federal

role had expanded beyond mostly fiscal support to include support for scientific and medical review of candidate conditions through ACHDNC and attention to IT needs. The need for longitudinal clinical information on infants identified and diagnosed by NBS as a source of data on clinical validity and utility was growing leading to recognition of a need to further address the development and standardization of EHRs in the U.S. However, there was little movement in developing robust health IT systems beyond the recognition of need. After significant NBS expansion in the 2000s, state budget constraints and competing public health emergencies (e.g., H1N1 flu, Zika, multiple hurricanes) slowed progress at the end of the 2010 -2019 decade [44,45] (Table S5).

**Table S5.** Conditions in NBS 2010-2019

| Years | Conditions in NBS |
|---|---|
| 2010s | Spinal Muscular Atrophy (SMA)<br>Pompe<br>Mucopolysaccharidosis I (MPS I)<br>Critical Cyanotic Congenital Heart Disease (CCHD)<br>X-linked adrenoleukodystrophy (X-ALD) |
| 2020s | None added to RUSP |

*4.1. Scientific Knowledge and Technology Expansion*

This decade saw a rapid advance of the development of new treatments and screening tools. The first gene therapy for a NBS condition, Zolgensma®, became available shortly after Spinraza® was cleared by FDA and led to the addition of SMA to NBS. In 2020, the first patient with SCD was treated using clustered regularly interspaced short palindromic repeats (CRISPR) Cas 9 gene replacement therapies. Even though these treatments were for rare disorders, concerns were raised about treatments becoming cost prohibitive as, for example, Zolgensma's® costs were approximately $2.2 million for the one-dose treatment.

Second-tier molecular or biochemical tests were being integrated within the NBS laboratories to better manage predictive values, reduce the costs of follow-up (false-positive screens) to families and to the health care system, and to inform treatment in emergent neonatal situations. Molecular NBS was moving from functional testing with minimal interpretation issues (e.g., TRECs) to germline testing to identify medically actionable targets. Sequencing arrays and gene panels that included many genes were increasingly a part of diagnostic evaluations. The National Human Genetics Research Institute (NHGRI)/NICHD-funded **N**ewborn **S**equencing **i**n **G**enomic Medicine and Public **H**eal**t**h (NSIGHT) program demonstrated the potential roles for exome sequencing (ES) or genome sequencing (GS) in NBS [46-48]. Among cases screening positively for an inborn error of metabolism (IEM) by traditional NBS methods across two study sites, specificity was 94% and 86% - 88% of newborns were detected (clinical sensitivity) by ES/GS [46]. Much of the reduction in clinical sensitivity resulted from the proportion of cases with variants of uncertain significance (VUS) that aren't reported in the screening results of asymptomatic people. Both study groups considered this performance to be inadequate to replace traditional NBS methods with ES/GS at this time. However, if other medically actionable screening biomarkers are not available for identification in the pediatric period, the value of ES/GS in identifying infants with variants in genes is apparent. For example, the potential for ES/GS to contribute to NBS for non-IEM disorders would enhance is apparent in NBS for early onset HL [49]. The number of infants with HL not detected at birth, but likely to realize a benefit from early treatment similar to that detected by NBS audiometry is nearly equal to the number of infants found by traditional HL NBS [49].

Simultaneous with the NSIGHT projects, NHGRI and NICHD funded The Clinical Genome Resource (ClinGen) that prioritized NBS genes among its variant curation activities to minimize uncertain findings. Genomic screening emerged through consented reporting of medically actionable secondary

findings (SF) [50] that could be screened over the lifespan, including in NBS. More recently, metabolomic profiles (the complete set of small-molecule (<1.5 kDa) metabolites) have shown potential for IEM screening in NBS [51].

Alternative approaches to broadening access to NBS outside of the public health system also emerged. The New York Screen Plus pilot study program funded by NIH [52] provides an example of an academic research program partnered with a state NBS laboratory to manage consented pilots of 5 lysosomal storage diseases (LSDs) (now expanded to 11 IEMs). Other approaches to broadening NBS have incorporated hospital-based screening as reported by Parad et al for DMD [53].

***4.2.*** *Government Public Policy and Public Health Role*

Although new treatment modalities were being developed, access to long-standing treatments such as medical foods for children with PKU and other inborn errors of metabolism (IEM) remained uneven even though ACHDNC was advocating for policies that ensured uniform access to the treatments, as access to treatment was key to a condition being added to the RUSP [54]. The Affordable Care Act of 2010 (ACA) requires health plans to cover screening for the federal RUSP with no cost sharing. However, treatment coverage for conditions on the RUSP was not addressed specifically in the ACA. In addition, state NBS programs continue to vary in both the number of mandated tests and their funding mechanisms for the NBS program, using a combination of state laboratory fees, third-party billing, and other federal and state funding [55,56]. NBS programs are uniquely funded among public health programs using funds from the U.S. Public Health Service Act, the Social Security Act, and NBS program fees to pay for the laboratory screening. States may also address costs with Medicaid and state general revenue to ensure universal access to screening, diagnosis, and treatment for all newborns. Additional funding may come from hospitals in the form of purchase of the filter cards used for collecting the blood samples. Others seek reimbursement through payers of health care costs.

NBS mandates and Medicaid rolled out state-by-state in the 1960s, 70s, and 80s, and are considered significant programs that improved infant health in the latter half of the 20$^{th}$ Century. Analysis of birth and death data over the period of institution of Medicaid and timing of NBS mandates show that NBS is associated with improvements in infant mortality in states with Medicaid. And in contrast, NBS mandates were not linked to significant declines in infant mortality in states without Medicaid [57].

Currently, depending on the state and Medicaid coverage, patients and families may have to assume costs for the nutrition and metabolic foods required to treat the disease, particularly given considerable variation in coverage policies in State insurance programs. Limiting reimbursement to the costs of screening tests alone undermines the common practice of using screening charges to fund follow-up services, counseling, and medical food or formula, particularly for low-income families [58]. The Medical Nutrition Equity Act was introduced in 2013 to address treatments for IEM. It has since been reintroduced but not yet authorized. The Act would require all private insurance plans (state regulated or self-insured/self-funded) and federal health programs, including Children's Health Insurance Program, Tricare, Medicaid, Medicare, and Federal Employee Health Benefit Plans to provide coverage for formula and low protein modified foods for all children and adults with IEM [57].

The 2010 - 2019 decade ended with continued discussion of the NBSSLA. Congress renewed NBSSLA in 2014 with a new requirement imposing consent requirements on NBS pilot studies. NBSSLA of 2019 passed the House in July 2021, but to date, the Senate has not yet held a mark-up of the bill due to renewed insistence that the bill require parents/guardians to opt-in for research using their newborn's unidentified DBS. Such a requirement would likely create a detrimental burden for rare disease research similar to the requirement imposed in 2014. Following a public comment period in 2017, HHS recognized the harm this policy could do to rare research and reversed the decision.

**References**


1. Bickel, H.; Gerrard, J.; Hickmans, E.M. Influence of phenylalanine intake on phenylketonuria. *Lancet* **1953**, 265, 812-813.
2. Green, A. The first treatment for PKU: The pioneers – Birmingham 1951. *Int J Neonatal Screen* **2021**; 7, 19: 1-15. https://doi.org/10.3390/ijns7010019.
3. Guthrie, R. Blood screening for phenylketonuria (letter to the Journal) *JAMA* **1961**; 178: 863.
4. Guthrie, R.; Susi A. A simple phenylalanine method for detecting phenylketonuria in large populations of newborn infants. *Pediatr* **1963**; 32: 338-43.
5. Guthrie, R.; Whitney, A. Phenylketonuria, Detection in the Newborn Infant as a Routine Hospital Procedure. U.S. Dept. Health, Education and Welfare, Sept. 30, **1964**
6. Hill, A,; Casey, R.; Zaleski, W.A. Difficulties and pitfalls in the interpretation of screening tests for the detection of inborn errors of metabolism. *Clin. Chim. Acta.* **1976**, 72:1-15.
7. Delaware Regulations established under Title 16 Delaware Code Sec. 122 (1) and Sec. 122 (3)(h) and Title 29 Delaware Code Section 7904. Available at: https://delcode.delaware.gov/title16/title16.pdf. Accessed 23 January 2022.
8. Committee for the Study of Inborn Errors of Metabolism: *Genetic screening: programs, principles and research*. Washington, DC: National Academy of Sciences; **1975**.
9. Therrell, B.L. Jr. Automation and computerization in newborn screening. In *Laboratory Methods in Neonatal Screening*, Editor Therrell BL. Publisher: Washington, DC, USA: American Public Health Association, **1993**; pp. 1-22.
10. Dussault, J.H., Laberge, C. Thyroxine ($T_4$) determination in dried blood by radioimmunoassay: a screening method for neonatal hypothyroidism. *Union Med Can* **1973**; 102: 2062–2064.
11. Dussault JH, Coulombe P, Laberge C, et al. Preliminary report on a mass screening program for neonatal hypothyroidism. *J Pediatr.* **1975**: 86;670–674.
12. Kan, Y.W.; Golbus, M.S.; Trecartin, R. Prenatal diagnosis of sickle cell anemia. *New Engl J Med.* **1976**; 294: 1039–1040.
13. Kan, Y.W.; Golbus, M.S.; Trecartin, R.F.; Filly, R.A.; Valenti, C.; Furbetta, M.; Cao, A. Prenatal diagnosis of beta thalassemia and sickle-cell anemia: experience with 24 cases. *Lancet.* **1977**; 1: 269–271.
14. Online Mendelian Inheritance in Man. Available online: https://www.omim.org (accessed on 16 December 2021).
15. Millington, D.S.; Terada, N.; Kodo, K.; Chace, D.H. A review: carnitine and acylcarnitine analysis in the diagnosis of metabolic diseases: advantages of tandem mass spectrometry. In: *Advances in chemical diagnosis and treatment of metabolic disorders,* Editor: Matsumoto, I. Publisher: New York: John Wiley & Sons, **1992**; 1: 59 –71.
16. Millington, D.S.; Kodo, N.; Norwood, D.L.; Roe, C.R. Tandem mass spectrometry: a new method for acylcarnitine profiling with potential for neonatal screening for inborn errors of metabolism. *J Inherit Metab Dis*. **1990**; 13: 321-4. doi: 10.1007/BF01799385. PMID: 2122093.
17. Chace, D.H.; Millington, D.S.; Terada, N.; Kahler, S.G.; Roe, C.R.; Hofman, L.F. Rapid diagnosis of phenylketonuria by quantitative analysis for phenylalanine and tyrosine in neonatal blood spots by tandem mass spectrometry. *Clin Chem* **1993**; 39; 66 –71.

18. Chace, D.; Naylor E. Expansion of newborn screening programs using automated tandem mass spectrometry. *Ment Retard Dev Disabil Res Rev* **1999**; 5: 150-4.
19. Hannon, W.H.; Grosse, S.D.; Therrell, B.L.; Becker, W.J.; Chace, D.H.; Cunningham, G.C.; Grady, G.F.; Hoffman, G.L.; Mann, M.Y.; Muenzer, J.; Mulvihill, J.J.; Panny SR. Using tandem mass spectrometry for metabolic screening among newborns. *MMWR* **2001**; 50/RR3: 1-34.
20. Zytkovicz, T.H.; Fitzgerald, E.F.; Marsden, D.; Larson, C.A.; Shih, V.E.; Johnson, D.M.; Strauss, A.W.; Comeau, A.M.; Eaton, R.B.; Grady, G.F. Tandem mass spectrometric analysis for amino, organic, and fatty acid disorders in newborn dried blood spots: a two-year summary from the New England Newborn Screening Program *Clin Chem* **2001**; 47: 1945-55.
21. U.S. Department of Health, Education and Welfare. PKU Blood Screening in Hospitals. Washington, D.C.: U.S. Dept. Health, Education, and Welfare; **1964**.
22. Wilson, J.M., and G. Jungner. Principles and Practice of Screening for Disease. (Public Health Paper Number 34). Geneva: World Health Organization; **1968**.
23. Wethers D., Pearson H, and Gaston M. Newborn Screening for Sickle Cell Disease and Other Hemoglobinopathies *Pediatr* **1989**; 83: 813–814.
24. Newborn Screening Task Force, American Academy of Pediatrics. Serving the family from birth to the medical home—Newborn screening: A blueprint for the future. *Pediatr* **2000**; 106(Suppl): 383–427.
25. Institute of Medicine (US) Committee on Assessing Genetic Risks; Editors: Lori B. Andrews, Jane E. Fullarton, Neil A. Holtzman, and Arno G. Motulsky. Washington (DC): National Academies Press (US); 1994.
26. ACMG Newborn Screening Expert Group. Newborn Screening: Toward a Uniform Screening Panel and System (Watson MS, Mann MY, Lloyd-Puryear MA, Rinaldo P, Howell RR, eds.) *Genet Med* **2006**; 8: Suppl 1; 1S-252S.
27. https://www.cdc.gov/mmwr/preview/mmwrhtml/mm6121a2.htm.
28. Kwan, A.; Abraham, R.; Currier, R.; Brower, A.; Andruszewski, K.; Abbott, J.K.; Baker, M.; Ballow, M.; Bartoshesky, L.E.; Bonilla, F.A.; et al. Newborn screening for severe combined immunodeficiency in 11 screening programs in the United States. *JAMA* **2014**; 312: 729-38.
29. Chan, K.; Puck, J.M. Development of population-based newborn screening for severe combined immunodeficiency. *J Allergy Clin Immunol* **2005**; 115; 391-8.
30. Kemper, A.R.; Hwu, W.L.; Lloyd-Puryear, M.; Kishnani, P.S. Newborn screening for Pompe disease: synthesis of the evidence and development of screening recommendation. *Pediatr* **2007**; 120: e1327-34.
31. Scott, C.R.; Elliott, S.; Hong, X.; Huang, J.Y.; Kumar, A.B.; Yi, F.; Pendem, N.; Chennamaneni, NK.; Gelb MH. Newborn screening for mucopolysaccharidoses: Results of a pilot study with 100,000 dried blood spots. *J Pediatr* **2020**; 216: 204-207.
32. Kraszewski, J.N.; Kay, D.M.; Stevens, C.F.; Koval, C.; Haser, B.; Ortiz, V.; Albertorio, A.; Cohen, L.L.; Jain, R.; Andrew, S.P.; et al. Pilot study of population-based newborn screening for spinal muscular atrophy in New York state. *Genet Med* **2018**; 20: 608-613.

33. Hall, P.L.; Li, H.; Hagar, A.F.; Jerris, S.C.; Wittenauer, A.; Wilcox, W. Newborn screening for X-linked adrenoleukodystrophy in Georgia: Experiences from a pilot study screening 51,081 newborns. *Int J Neonatal Screen* **2020**; 6: 81-.
34. Lee, S.; Clinard, K.; Young, S.P., Rehder, C.W.; Fan, Z.; Calikoglu, A.S.; Bali, D.S.; Bailey, D.B. Jr.; Gehtland, L.M.; Millington, D.S.; et al. Evaluation of X-linked adrenoleukodystrophy newborn screening in North Carolina. *JAMA Netw Open* **2020**; e1920356.
35. Lloyd-Puryear, M.A.; Crawford, T.O.; Brower, A.; Stephenson, K.; Trotter, T.; Goldman, E.; Goldenberg, A.; Howell, R.R.; Kennedy, A.; Watson, M. Duchenne Muscular dystrophy newborn screening, a case study for examining ethical and legal issues for pilots for emerging disorders: considerations and recommendations. *Int J Neonatal Screen* **2018**; 4; 6-.
36. Therrell, B.L.; Hannon, W.H.; National evaluation of U.S. newborn screening system components. *Ment Retard Dev Disabil Res Rev* **2006**; 12: 236-45.
37. Government Accountability Office. Newborn Screening: Characteristics of State Programs GAO-03-449 Published: 17 Mar 2003. Publicly Released: Apr 16, **2003**. Newborn Screening: Characteristics of State Programs GAO-03-449 Published: Mar 17, 2003. Publicly Released: Apr 16, 2003.
38. Saarlas, K.N.; Hinman, A.R.; Ross, D.A.; Watson, W.C. Jr.; Wild, E.L.; Hastings, T.M.; Richmond, P.A. All Kids Count 1991-2004: developing information systems to improve child health and the delivery of immunizations and preventive services. *J Public Health Manag Pract*. **2004**; Suppl: S3-15. PMID: 15643355.
39. Calonge, N.; Green, N.S.; Rinaldo, P.; Lloyd-Puryear, M.; Dougherty, D.; Boyle, C.; Watson, M.; Trotter, T.; Terry, S.F.; Howell, R.R.; Advisory Committee on Heritable Disorders in Newborns and Children. Committee report: Method for evaluation conditions nominated for population-based screening of newborns and children. *Genet Med*. **2010**; 12: 153-9.
40. Linzer, D.S.; Lloyd-Puryear, M.A.; Mann, M.; Kogan, M.D. Evolution of a child health profile initiative. *J Public Health Manag Pract*. **2004**; Suppl:S16-23. Doi: 10.1097/00124784-200411001-00003. PMID: 15643353.
41. Hinman, A.R.; Atkinson, D.; Diehn, T.N.; Eichwald, J.; Heberer, J.; Hoyle, T.; King, P.; Kossack, R.E.; Williams, D.C.; Zimmerman, A. Principles and core functions of integrated child health information systems. *J Public Health Manag Pract*. **2004**; Suppl:S52-6. Doi: 10.1097/00124784-200411001-00008. PMID: 15643359.
42. Abhyankar, S.; Lloyd-Puryear, M.A.; Goodwin, R.; Copeland, S.; Eichwald, J.; Therrell, B.L.; Zuckerman, A.; Downing, G.; McDonald, C.J. Standardizing newborn screening results for health information exchange. American Medical Informatics *Association (AMIA) Symposium Proceedings* **2010**; 1-5.
43. National Center for Biomedical Information. Available at: https://lhncbc.nlm.nih.gov/newbornscreeningcodes/ Accessed 16 December 2021.
44. Alfonso, Y.N.; Leider, J.P.; Resnick, B.; McCullough, J.M.; Bishai, D. US public health neglected: flat or declining spending left states ill equipped to respond to COVID-19. *Health Aff* (Millwood) **2021**; 40: 664-71.

45. Baker, M.; Ivory, D. Why public health faces a crisis across the U.S. The New York Times, October 18, **2021** (https://www. nytimes. com/ 2021/ 10/ 18/ us/ coronavirus - public - health.htm.
46. Berg, J.S.; Agrawal, P.B.; Bailey, D.B.; Beggs, A.H.; Brenner, S.E.; Brower, A.M.; Butler, I.; Cakici, J.; Ceyhan-Birsoy, O.; Chan, K,; et al. **N**ewborn **S**equencing **I**n **G**enomic medicine and public **H**eal**T**h (NSIGHT). *Pediatr* **2017**; 139: 2016-2022.
47. Holm, I.A.; Agrawal, P.B.; Ceyhan-Birsoy, O.; Christensen, K.D.; Fayer, S.; Frankel, L.A.; Genetti CA, Krier JB, LaMay RC, Levy HL, et al.. The BabySeq project: implementing genomic sequencing in newborns. *BMC Pediatr* **2018**; 18: 225- .
48. Adhikari, A.N.; Gallagher, R.C.; Wang, Y.; Currier, R.J.; Amatuni, G.; Bassaganyas, L.; Chen, F.; Kundu, K.; Kvale, M.; Mooney, S.D.; et al. The role of exome sequencing in newborn screening for inborn errors of metabolism. *Nat Med* **2020**; 26: 1392-1397.
49. Shearer, A.E.; Shen, J.; Amr, S.; Morton, C.C.; Smith, R.J.; A proposal for comprehensive newborn hearing screening to improve identification of deaf and hard-of-hearing children. Newborn Hearing Screening Working Group of the National Coordinating Center for the Regional Genetics Networks. *Genet Med* **2019**; 21: 2614-2630.
50. Kalia, S.S.; Adelman, K.; Bale, S.J.; Chung, W.K.; Eng, C.; Evans, J.P.; Herman, G.E.; Hufnagel, S.B.; Klein, T.E.; Korf, B.R.; et al; on behalf of the ACMG Secondary Findings Maintenance Working Group. *Genet Med* **2017**; 19: 249-55.
51. Liu, N.; Xiao, J.; Gijavanekar, C.; Pappan, K.L.; Glinton, K.E.; Shayota, B.J.; Kennedy, A.D.; Sun, Q.; Sutton, V.R.; Elsea, SH. Comparison of Untargeted Metabolomic Profiling vs Traditional Metabolic Screening to Identify Inborn Errors of Metabolism. *JAMA Netw Open*. **2021**; 4: e2114155.
52. Wasserstein, M.P.; Caggana, M.; Bailey, S.M.; Desnick, R.J.; Edelman, L.; Esxtrella, L.; Holzman, I.; Kelly, N.R.; Kornreich R.; Kupchik,; et al. The New York pilot newborn screening program for lysosomal storage diseases: Report of the First 65,000 Infants. *Genet Med* **2019**; 21: 631-640.
53. Parad, R.B.; Sheldon, Y.; Bhattacharjee, A. Implementation of Hospital-Based Supplemental Duchenne Muscular Dystrophy Newborn Screening (sDMDNBS): A Pathway to Broadening Adoption. *International Journal of Neonatal Screening*. **2021**; 7: 77.
54. Berry, S.A.; Brown, C.S.; Greene, C.; Camp, K.M.; McDonough, S.; Bocchini, J.A.; Follow-up and Treatment Workgroup for the Advisory Committee on Heritable Disorders in Newborns and Children. Medical foods for inborn errors of metabolism: history, current status, and critical need. Pediatr **2020**; 145: 2019-20.
55. Therrell, B.L.; Williams, D.; Johnson, K.; Lloyd-Puryear, M.A.; Mann, M.Y.; Ramos, L.R. Financing newborn screening: sources, issues, and future considerations. *J Public Health Manag Pract* **2007**; 13: 207-13.
56. Johnson, K.; Lloyd-Puryear, M.A.; Mann, M.Y.; Ramos, L.R.; Therrell, B.L. Financing state newborn screening programs: sources and uses of funds. *Pediatr* **2006**; 117: 270-279.
57. Sohn H and Timmermans R, Inequities in newborn screening: Race and the role of Medicaid, *Science Direct Population Health* **2019**; (9), December 2019, 100496

58. U.S. Congress. Available online: https://www.congress.gov/bill/117th-congress/house-bill/3783?s=1&r=55#:~:text=This%20bill%20expands%20coverage%20under,and%20metabolic%20disorders%20and%20conditions. Accessed on 23 January 2022.
